# Short-term artificial adaptation of *Rhizoglomus irregulare* to high phosphate levels and its implications for fungal-plant interactions: phenotypic and transcriptomic insights

**DOI:** 10.3389/fpls.2024.1385245

**Published:** 2024-04-23

**Authors:** Eva Lucic-Mercy, Louis Mercy, Andrea Jeschke, Carolin Schneider, Philipp Franken

**Affiliations:** ^1^ INOQ GmbH, Schnega, Germany; ^2^ Institute of Microbiology, Friedrich Schiller University, Jena, Germany; ^3^ Erfurt Research Centre for Horticultural Crops, University of Applied Sciences Erfurt, Erfurt, Germany

**Keywords:** phosphate, mycorrhizal symbiosis, short-term adaptation, *Rhizoglomus irregulare*, biotechnology

## Abstract

Arbuscular mycorrhizal fungi (AMF) play a crucial role in enhancing plant growth, but their use in agriculture is limited due to several constraints. Elevated soil phosphate levels resulting from fertilization practices strongly inhibit fungal development and reduce mycorrhizal growth response. Here, we investigated the possibility of adapting *Rhizoglomus irregulare* to high phosphate (Pi) levels to improve its tolerance. A fungal inoculum was produced through multiple generations in the presence of elevated Pi and used to inoculate melon plants grown under low and high phosphate conditions. Our results revealed distinct phenotypic and transcriptomic profiles between the adapted and non-adapted *Rhizoglomus irregulare*. The Pi adapted phenotype led to enhanced root colonization under high Pi conditions, increased vesicle abundance, and higher plant biomass at both phosphate levels. Additionally, the adaptation status influenced the expression of several genes involved in Pi uptake, Pi signaling, and mitochondrial respiration in both symbiotic partners. While the underlying mechanisms of the adaptation process require further investigation, our study raises intriguing questions. Do naturally occurring phosphate-tolerant AMF already exist? How might the production and use of artificially produced inocula bias our understanding? Our findings shed light on the adaptive capacities of Glomeromycota and challenge previous models suggesting that plants control mycorrhizal fungal growth. Moreover, our work pave the way for the development of innovative biotechnological tools to enhance the efficacy of mycorrhizal inoculum products under practical conditions with high phosphate fertilization.

## 1 Introduction

Arbuscular mycorrhizal fungi (AMF) are a widespread class of fungi forming mutualistic symbioses with most (72%) plant families on earth (Brundrett and Tedersoo, 2018). These microorganisms, belonging to Glomeromycota (Tedersoo et al., 2018), have been shown (i) to improve mineral plant nutrition (macro and micronutrients), (ii) to increase plant tolerance and resistance against abiotic and biotic stresses, (iii) to regulate plant development (i.e. rooting, flowering, fruit and seed formation), (iv) to improve biological and physical soil properties (Bennett and Meek, 2020; Singh et al., 2022; Shi et al., 2023). In turn, plant hosts provide a physical and physiological habitat associated with delivering of different energy sources that allows AM fungi to complete their biotrophic life cycle (Rich et al., 2017; Bedini et al., 2018).

These benefits to plants led to classify mycorrhizal fungi products as biostimulants (Rouphael et al., 2015), although they can also be considered as bioprotectants (Xavier and Boyetchko, 2002). However, despite the recognized potential of AMF as ecosystem service tools (Gianinazzi et al., 2010), challenges remain under practical conditions when considering the fertilization and/or soil chemical properties under conventional farming (Vosátka et al., 2008; Ijdo et al., 2011; Berruti et al., 2016). Mycorrhizal fungi are widespread in almost all soil ecotypes and harbor a high plasticity at species level, but their fitness is often impeded in presence of high available phosphate (Pi) concentrations. This nutrient can reduce severely mycorrhizal extra- and intra-radicular mycelial development, as well as arbuscule abundance and function (Olsson et al., 2002; Smith and Read, 2008; Kobae and Hata, 2010; Balzergue et al., 2013). The negatively correlated responses between phosphate concentration in soil and mycorrhizal colonization and functions are well investigated (Smith and Read, 2008). Physiological models were proposed that integrate hormonal signaling with plant and fungal metabolism that occur upon two different context of phosphate concentrations (Bedini et al., 2018). However, there is an urgent need to define innovative strategies to improve mycorrhizal development and performance under high phosphate levels as they appear under practice conditions in soils and substrates.

AMF face constant environmental fluctuations, including various biotic and abiotic stresses and changes in nutrient availability. Like in all organisms, environmental variation causes selection for different traits in AM fungi, which have the ability to rapidly produce variable progeny adapted to fluctuating stress conditions or host plant species (Angelard et al., 2014; Behm and Kiers, 2014; Millar and Bennett, 2016; Branco et al., 2022). It was also suggested that abiotic factors constitute potentially greater selection pressure than host plants for local adaptation of AMF (Helgason and Fitter, 2009) and that they can adapt to abiotic stress independently of their host plant (Millar and Bennett, 2016). Locally adapted traits can also be artificially induced, leading to improved mycorrhizal plant response as shown for salt or heavy metals (Sharifi et al., 2007; Oliveira et al., 2010; Bui and Franken, 2018). It seems therefore that modifying tolerance to different elements is possible, but is, in a first sense, not obvious for phosphate, given the fact that the fungal colonization in dependance on phosphate availability seems to be under the control of the host plant (Shi et al., 2021; Wang et al., 2021; Das et al., 2022). Interestingly, the concept of adaptation is commonly associated with the notion of stress, whereas phosphate is not typically considered an abiotic stressor *per se*, unless its concentration reaches starvation levels. Instead, it is an essential element that contributes to the cellular redox and energy status (ATP, NADP) of both plants and fungi, and is therefore closely linked to the mitochondrial respiratory pathway, which plays an important role in mycorrhizal behavior. The cytochrome oxidase (COX) and alternative oxidase (AOX) mitochondrial pathways have been shown to modulate spore germination and hyphal branching during the presymbiotic phase (Campos et al., 2015; Mercy et al., 2017). The transcription patterns of these genes have been associated with distinct intraradical mycorrhizal phenotypes in potato (Mercy et al., 2017). AOX is considered as an element of flexibility in fungal metabolism, allowing acclimation to different stress conditions and ecological fitness of fungi (Grahl et al., 2012; Thomazella et al., 2012; Xu et al., 2012; Campos et al., 2015; Del-Saz et al., 2018). This ascribed role of AOX in fungi is also true for plants (Arnholdt-Schmitt et al., 2006; Saha et al., 2016; Selinski et al., 2018), and in addition, it plays an important role in seed dormancy and germination (Mohanapriya et al., 2019; Yu et al., 2021; Racca et al., 2022). To complete this picture, adaptation to phosphate should also consider the intricate interplay of phosphate transporters (PTs) and phosphate signaling genes in both fungi and plants, which also play a role in controlling symbiotic dynamics and function. In fungi, at least 9 PT isoforms have been characterized in *R. irregulare*, expressed in both intra- and extra-radical mycelium (Benedetto et al., 2005; Balestrini et al., 2007; Tisserant et al., 2012; Fiorilli et al., 2013; Walder et al., 2016; Mercy et al., 2017; Zhou et al., 2021; Xie et al., 2022). Furthermore, the fungal phosphate homeostasis is regulated by the PHO pathway, and the recent expansion of sequenced Glomeromycota genomes has revealed that AMF possess many key genes involved in this pathway (Tisserant et al., 2012; Zhou et al., 2021), some of which have been shown to be significantly regulated in mycorrhizal roots in response to phosphate (Zhou et al., 2021; Zhang et al., 2023). From the plant side, host plants possess two possible Pi uptake routes: the direct uptake pathway (DUP), mediated by non-mycorrhiza-regulated Pht1 members, and the mycorrhizal uptake pathway (MUP), facilitated by AM-induced or AM-specific PTs (Bucher, 2007; Javot et al., 2007; Ferrol et al., 2019). Notably, many members of the PHT1 family are transcriptomically up-regulated in phosphate-depleted plants and repressed by higher phosphate concentrations (Nussaume et al., 2011).

Several studies pointed that if the formation and functionality of arbuscular mycorrhizal symbiosis is adversely affected by high Pi in pot experiments under controlled conditions, the influence of Pi fertilizer on AMF community structure and abundance is variable under field conditions. Some studies claimed that high soil Pi supply does not always have a negative impact on AMF diversity, fungal colonization or performance of inoculated crops (Jansa et al., 2014; Liu et al., 2016; Higo et al., 2020; Peña Venegas et al., 2021; Lutz et al., 2023). These works pointed complex influences of soil phosphate on AMF and suggested that the response to this element may be related to various factors and notably AMF identity. Liu et al. (2016) suggested that the indigenous AMF community may be selected towards specific taxa or strains that are strong competitors and less sensitive to Pi fertilizers. This raises the question if Pi-tolerant AMF lines do exist naturally or might occur in managed conventional systems. However, the demonstration that such Pi-tolerant AM species would exist in field conditions is challenging, since their isolation and testing should consider the myriad of interactions that can occur within a soil and the environment, as the variable presence of other elements like N, the microbiome, or different host plant genotypes. Therefore, as first step, it seems more reasonable to obtain proof of concept under controlled condition.

Based on these considerations, we investigated the following hypotheses: (1) the induction of artificial adaptation in *Rhizoglomus irregulare* (syn. *Rhizophagus irregularis*) can enhance tolerance to high phosphate levels, (2) such adaptation results in phenotypic changes in both the fungal symbiont and the host plant, and (3) these variations are accompanied by transcriptomic changes in both organisms.

To address these research questions, *R. irregulare* was cultivated in monoxenic *in vitro* system for five generations in the presence or absence of high phosphate concentrations. Subsequently, the fungi were cultivated under greenhouse conditions for one season, maintaining two different phosphate conditions. This approach yielded two *ex vitro* inocula containing *R. irregulare* with non-tolerant (RiQS81-Pi^-^) and tolerant (RiQS81-Pi^+^) traits towards high Pi. These inocula were then used in a greenhouse assay involving melon plants, which were grown under high Pi (HP) and low Pi (LP) conditions. We then assessed several fungal and plant phenotypic parameters, as well as the transcription of several genes involved in respiration, Pi signaling and transport, accompanied by several other symbiotic markers. We observed significant differences between the two *R. irregulare* inocula at both phenotypic and transcriptomic levels. The implications of these findings are discussed in terms of physiology, ecology, research models, and inoculum production.

## 2 Materials and methods

### 2.1 Fungal material

The fungal inocula used in this trial were produced by using *in vitro* spores obtained from two distinct cultural itineraries (Bedini, Varela et al., in preparation) as starter material. Briefly, *in vitro Rhizoglomus irregulare* (the homokaryotic INOQ strain QS81) cultures were set up from a single initial plate as described in Declerck et al. (1998), on normal MSR medium or on MSR medium containing 100 ppm phosphorus (P; 3,23 mM KH_2_PO_4_). Several dozen spores were transferred on each new plate, to reduce the potential variation bias a single spore could bring. This process was repeated iteratively for 5 generations, and 100 *in vitro* spores of the 5^th^ generation of both culture protocols, with or without addition of phosphate, were then used to inoculate *Trifolium pratensis* pots in greenhouse. The plants were grown while maintaining consistent cultural conditions to the *in vitro* ones, with low (10 ppm P) and high (100 ppm P) phosphate concentrations, applied in the form of KH_2_PO_4_ mixed directly in the substrate (respectively 43.93 mg/L and 439.37mg/L). Fertilization was performed weekly using a standard Hoagland solution (Hoagland and Arnon, 1938) devoid of phosphate, and both fertilization and watering were carefully applied to prevent any leaching at the bottom of the pots. After 6 months, the colonized root systems were harvested, homogenized, and filtered through a 425 µm mesh sieve to obtain first *ex vitro* powder inocula, corresponding overall to the 6^th^ generation. The two fungal cultures were designated as “Pi^-^ phenotype” (RiQS81-Pi^-^) and “Pi^+^ phenotype (RiQS81-Pi^+^), referring to the presence or absence of high phosphate during the successive propagations. Before subsequent use in the trial, the identity of RiQS81-Pi^+^ and RiQS81-Pi^-^ *R. irregulare* was checked by sequencing of the FLR2-LROR fragment and by RFLP on mitochondrial rDNA (**Supplementary Material 1.1**; **Supplementary Figure S1**) to confirm that the same isolate was present in both inocula.

### 2.2 Plant growth conditions

A greenhouse experiment was implemented using 1 L plastic pots filled with pure sterilized sand (2 x 6 h at 121°C) containing 10 ppm P (43.93 mg KH_2_PO_4_ per L substrate, also noted as LP for low Pi concentration) or 100 ppm P (439.37mg KH_2_PO_4_ per L substrate, also noted as HP for high Pi concentration). KH_2_PO_4_ was mixed in the substrate as powder form and care was taken that no leaching occurs from the bottom of the pot during subsequent waterings. Plants were inoculated or not with 100 mg *ex vitro* inoculum powder per L substrate, mixed in the substrate. Then, melon seeds (*Cucumis melo* cv Charantaise) were placed in the pot (1 seed per pot) according to the following conditions (completely randomized design): (i) non-inoculated plants, 10 ppm Pi (NM LP); (ii) plants inoculated with RiQS81-Pi^-^ inoculum, 10 ppm Pi (RiQS81-Pi^-^ LP); (iii) plants inoculated with RiQS81-Pi^-^ inoculum, 100 ppm Pi (RiQS81-Pi^+^ HP); (iv) non inoculated plants, 100 ppm Pi (NM HP); (v) plants inoculated with RiQS81-Pi^+^ inoculum, 10 ppm Pi (RiQS81-Pi^+^ HP); (vi) plants inoculated with RiQS81-Pi^+^ inoculum, 100 ppm Pi (RiQS81-Pi^+^ HP). 8 replicates were implemented for each of the 6 conditions. Plants were fertilized (once per week, 80 ml per pot) with Hoagland solution without Pi.

Plants were harvested at day 77 after inoculation (DAI). Shoot and root fresh biomasses were assessed, and 1 g of fresh roots was immediately frozen in liquid nitrogen for molecular analyses. Remaining roots systems were stained with black ink (modified from Vierheilig et al., 1998, **Supplementary Material 1.2**) and fungal alkaline phosphatase (ALP) stain (modified from Guillemin et al., 1995; **Supplementary Material 1.3**). Fungal colonization patterns were assessed according to a modified protocol from Trouvelot et al., 1986. Two Excel spreadsheet versions were developed to facilitate the analysis, one including the classic fungal parameters F%, M%, m%, A% and a% (Mycocheck classic; **Supplementary Spreadsheet 1**) and one including new fungal parameters compared to the original version (Mycocheck advanced; **Supplementary Spreadsheet 2**). The added parameters include vesicle abundance in the root system and in colonized root fragments (V%; v%), intraradical hyphal abundance in the root system and in colonized root fragments (H%; h%), and the Arum/Paris ratio. Parameters considering the relative proportion of each intraradical structure within the roots were also included. Indeed, the evaluation of the score of each fungal structure is done independently from the other fungal parameters, which introduces a bias about the real abundance of the evaluated structures in the colonized roots. Therefore, we also calculate A^r^%, V^r^% and H^r^%, which are the relative proportions of arbuscules, vesicles and intraradical mycelium, respectively, in the whole root system. These parameters can then be used to evaluate the abundance of arbuscules, vesicles and intraradical mycelium within the M% (a^r^%, v^r^%, h^r^%). The formulas are detailed in **Supplementary Spreadsheet 2**.

Samples from shoot parts were harvested, dried, and dried leaf material was used for analyzing the Pi content of melon leaves (g/kg DW) according to the DIN EN 15621:2017-10 norm (Landesamt für Landwirtschaft und Ländlichen Raum, Thüringen, DE).

### 2.3 Gene identification and expression analysis

The RNA accumulation of selected melon and *R. irregulare* genes were assessed in non-mycorrhizal and mycorrhizal roots inoculated with both strains (RiQS81-Pi^+^ and RiQS81-Pi^-^) and in two contrasting Pi conditions. Melon genes encoding Pi transporters of the Pht1 family, the alternative oxidase (AOX), the cytochrome C (CytC), two isoforms of the subunit 5b of the cytochrome oxidase (COX5b) which was described as the most conserved among nuclear-encoded subunits (Rizzuto et al., 1991; Grossman and Lomax, 1997), the lactate dehydrogenase (LDH), two mycorrhiza-responsive blue copper proteins (BCP1a and BCP1b), as well as proteins involved in the fatty acid metabolism and transport (FatM, RAM2, STR1 and STR2) were identified by comparison with known sequences from other plant species and retrieved from the CuGenDBv2 database for cucurbit genomics (Yu et al., 2023). Genes of interest from *R. irregulare*, involved in phosphate transport (PHT1 family) and signaling (PHO pathway), sugar transport (MST), as well as respiration (*COX5b*, *CytC* and *AOX*), were selected based on existing publications (Fiorilli et al., 2013; Mercy et al., 2017; Zhou et al., 2021; Xie et al., 2022). All accession numbers are listed in **Supplementary Table S1**.

Total RNA was extracted from 100 mg of melon roots using the RNeasy Plant Mini Kit (Qiagen, Hilden, Germany), according to manufacturer’s instructions. 300 ng of extracted RNA were reverse transcribed into cDNA using the RevertAid RT Kit (Thermo Fisher Scientific, Massachusetts, USA) in 20 μL following the supplier’s instruction. The cDNAs were 1:5 and 1:10 diluted (respectively for fungal and plant genes) prior to analysis. Relative expression levels of target genes were analyzed by qRT-PCR experiment using the CFX Connect Real-Time PCR Detection System (Bio-Rad, California, USA). Amplification reactions were prepared in 20 μl reaction volumes using the Blue S’Green qPCR master mix (Biozym, Hessisch Oldendorf, Germany). Eight independent biological replicates were analyzed per treatment and each sample was analyzed in triplicate. The specificity of the different amplicons was checked by a melting curve analysis at the end of the amplification protocol. Standard curves of dilution series from pooled cDNAs were used for PCR efficiency calculations for each primer pair. Several candidates were evaluated for further use as reference gene for normalization of the transcript data of target genes. After evaluation of expression stability using the applications BestKeeper, NormFinder and GeNorm (Vandesompele et al., 2002; Andersen et al., 2004; Pfaffl et al., 2004), *CmACT* and *CmADP* for the melon (Kong et al., 2014) and *GiICL* for *R. irregulare* (Lammers et al., 2001) were chosen as reference genes for our experimental conditions. Results were presented as means of normalized expression values (dCq=Cq_[GOI]-_Cq_[HKG]_). All primers are listed in **Supplementary Table S1**.

### 2.4 Data analysis

For all phenotypic and transcriptomic data (plant and fungus), statistical analyses were performed by multiple-comparison 2-ways ANOVA (post-hoc Tukey, with 2 factors as phosphate levels and mycorrhizal inoculation – respectively named phosphate and AMF). The statistical analyses were conducted with the SAS enterprise guide 9.4 (SAS Institute Inc., Cary, USA).

## 3 Results

### 3.1 The adaption process affected fungal colonization parameters and the plant growth

#### 3.1.1 The two *R. irregulare* Pi phenotypes impacted differentially the plant biomass

**Figure 1:**
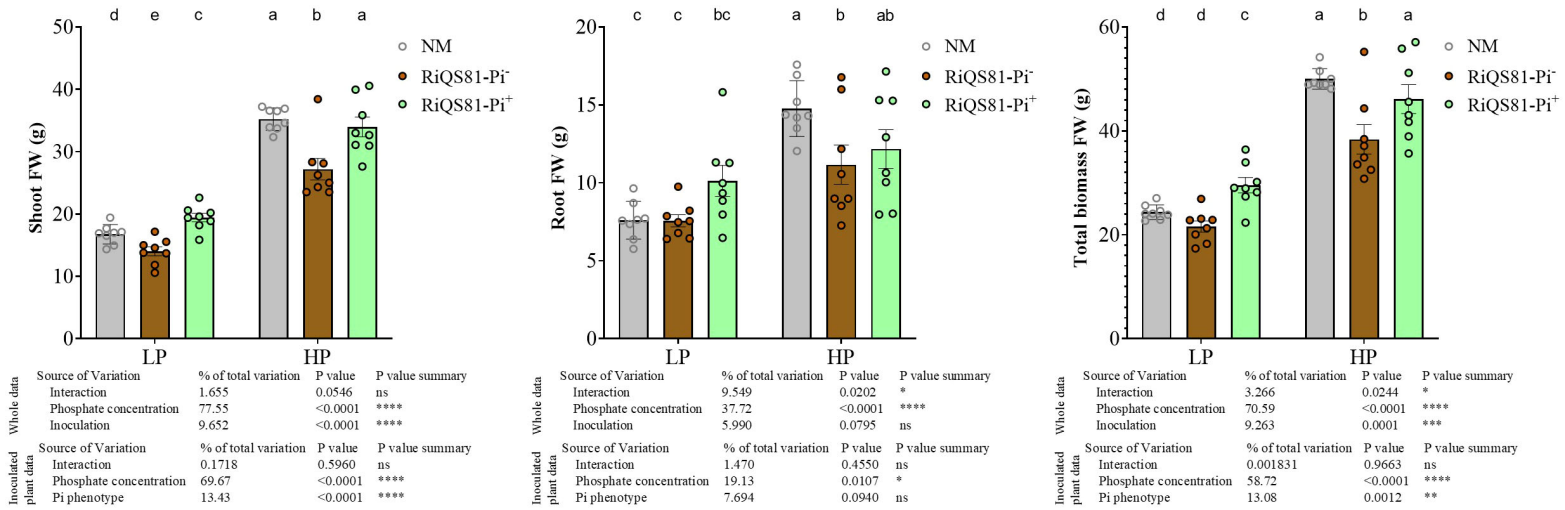
Effects of phosphate and inoculation on melon plant growth parameters. Melon plants (Cucumis melo) inoculated or not with RiQS81-Pi^-^/RiQS81-Pi^+^ *Rhizoglomus irregulare* inoculum, growing under two levels of phosphate (Pi). Plants were harvested 77 DAI. Data show means (*n* = 8) ± s.e.. Treatments sharing the same letter are not significantly different (*p* < 0.05 Tukey multiple-comparison ANOVA 2 ways, with assessment on the whole design and when considering inoculated plants alone). The tables provide statistical details per factor; significant differences are indicated by stars (ns: non-significant; * P < 0.05; ** P < 0.005;*** P < 0.0005; **** P < 0.00005). NM, Non-inoculated plants; HP, high Pi; LP, low Pi.

The plant phenotypic parameter data are shown **Figure 1**. The impact of phosphate as factor was significant overall, and the total fresh biomass, as well as shoot and root fresh weight were all increased at high Pi, as compared to low Pi. A significantly higher biomass (total fresh weight and shoot fresh weight) is observed for plants inoculated with RiQS81-Pi^+^ inoculum compared to those inoculated with the RiQS81-Pi^-^ one. The highest values of total biomass were obtained under high Pi with non-inoculated plants and plants inoculated with RiQS81-Pi^+^. Under high Pi, application of RiQS81-Pi^-^ *R. irregulare* significantly reduced the plant growth parameters compared to the non-inoculated plants. Under low P, and as compared to the non-inoculated plants, inoculation with RiQS81-Pi^-^ strain significantly reduced shoot biomass, but without significant effect on root and total biomass, whereas the RiQS81-Pi^+^ significantly promoted shoot and total biomass. In addition, shoot Pi content (**Supplementary Figure S2**) was only affected by Pi concentration, and not by mycorrhizal status, indicating that the nutrient use efficiency of the plant was not affected by either fungal inoculum.

#### 3.1.2 Fungal phenotype after black ink staining

**Figure 2:**
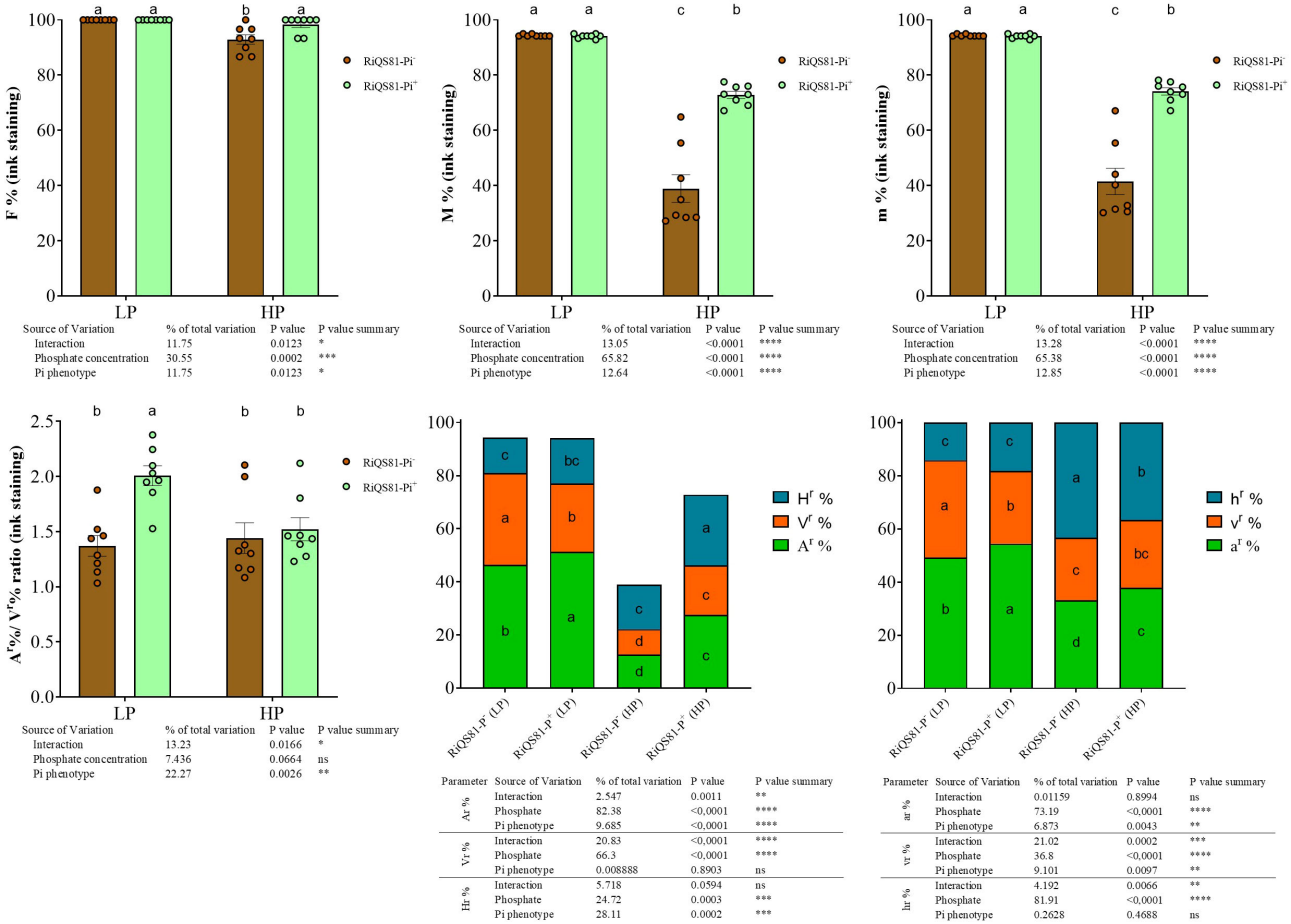
Fungal phenotypic parameters after black ink staining of RiQS81-Pi^-^ and RiQS81-Pi^+^ *R. irregulare* growing within melon (*Cucumis melo*) roots under two levels of Pi. Roots were harvested 77 DAI, stained and fungal parameter were evaluated. Data show means (*n* = 8) ± s.e. Treatments sharing the same letter are not significantly different (*p* < 0.05; Tukey multiple-comparison ANOVA 2 ways), the non-inoculated plants were considered as outgroup to control data normality. The tables provide statistical details per factor; significant differences are indicated by stars (ns, non-significant; * P < 0.05; ** P < 0.005;*** P < 0.0005;**** P < 0.00005). Data analyses were performed separately for each parameter. NM, Non-inoculated plants; HP, high phosphate; LP, low phosphate; F%, frequency of colonized root fragments; M%, intensity of mycorrhizal colonization in the whole root system; m%, intensity of the mycorrhizal colonization in the colonized root fragments; A^r^%, V^r^% and Hr^r^%: relative proportion of arbuscules, vesicles and intraradical mycelium respectively, in the whole root system; a^r^%, v^r^% and h^r^%, relative proportion of arbuscules, vesicles and intraradical mycelium respectively, within the M%.

Data obtained after the non-vital black ink staining are shown **Figure 2** and **Supplementary Figure S3**. The impact of phosphate was significant overall, with a decrease of most of fungal parameter values under high Pi conditions. The impact of AMF inoculum type was also significant overall, with differences due to the Pi levels. Under low Pi, plants inoculated with RiQS81-Pi^+^ presented a (i) significant increase of the arbuscule parameters (A^r^%, a^r^% and A^r^%/V^r^% ratio); (ii) significant reduction of the vesicle parameters (v%, V%, V^r^% and v^r^%) and (iii) no significant influence for all other investigated fungal parameters. Under high Pi, as compared to the RiQS81-Pi^-^ inoculum, the RiQS81-Pi^+^ (i) significantly increased F%, M%, m%, A%, V%, H%, A^r^%, V^r^%, H^r^%, a^r^%, h^r^%, and *Arum*/*Paris* ratio, (ii) significantly decreased the h% and (iii) had no significant effect on all other fungal parameters studied.

#### 3.1.3 Fungal phenotype after ALP staining

**Figure 3:**
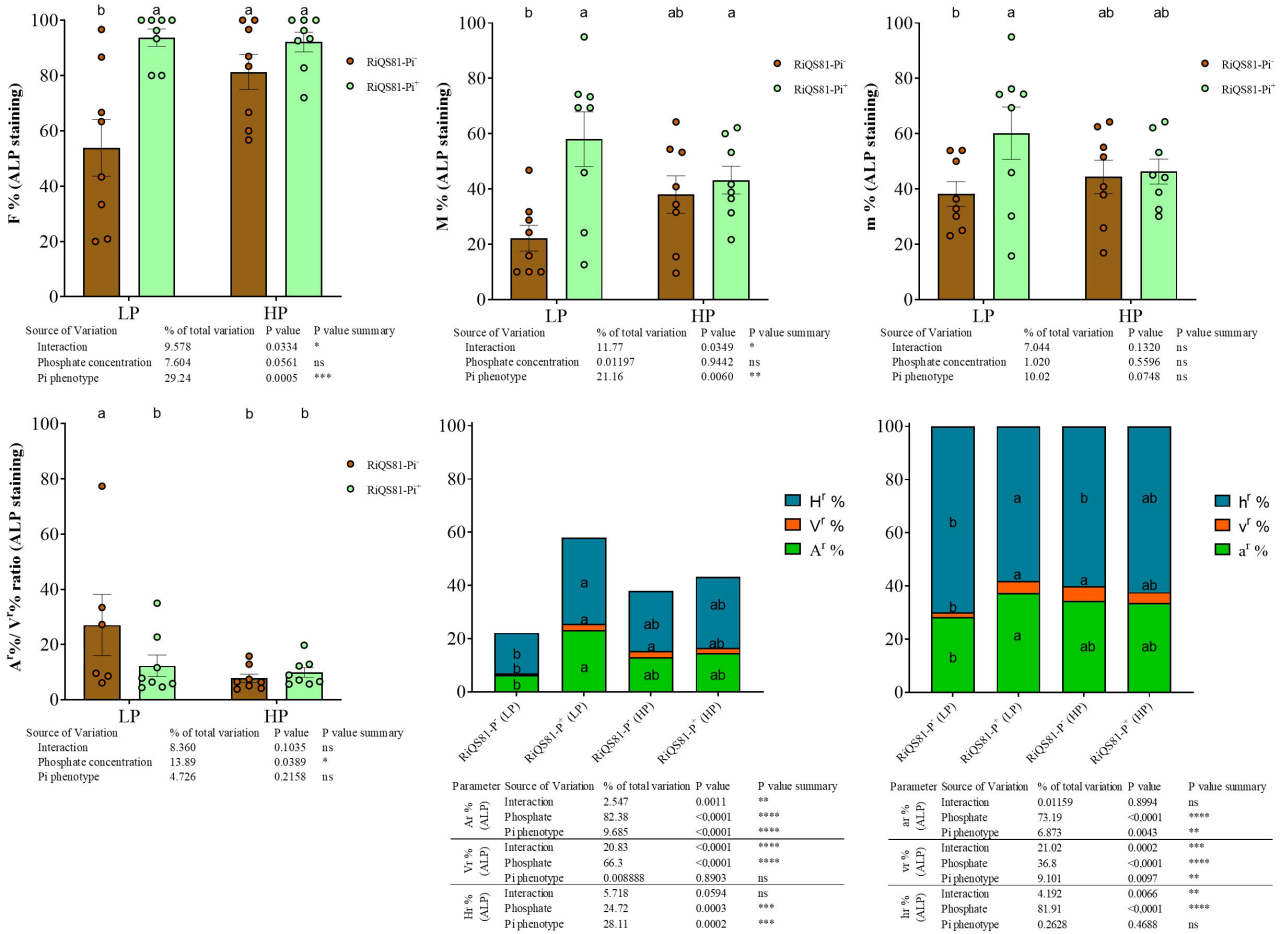
Fungal phenotypic parameters after ALP staining of RiQS81-Pi^-^ and RiQS81-Pi^+^ *R. irregulare* growing within melon (*Cucumis melo*) roots under two levels of Pi. Roots were harvested 77 DAI and stained for alkaline phosphatase activity. Data show means (*n* = 8) ± s.e. Treatments sharing the same letter are not significantly different (*P* < 0.05; Tukey multiple-comparison ANOVA 2 ways), the non-inoculated plants were considered as outgroup to control data normality. The tables provide statistical details per factor; significant differences are indicated by stars (ns: non-significant; * P < 0.05; ** P < 0.005;*** P < 0.0005; **** P < 0.00005). Data analyses were performed separately for each parameter. NM, Non-inoculated plants; HP, high phosphate; LP, low phosphate; F%, frequency of colonized root fragments; M%, intensity of mycorrhizal colonization in the whole root system; m%, intensity of the mycorrhizal colonization in the colonized root fragments; A^r^%, V^r^% and Hr^r^%: relative proportion of arbuscules, vesicles and intraradical mycelium respectively, in the whole root system; a^r^%, v^r^% and h^r^%: relative proportion of arbuscules, vesicles and intraradical mycelium respectively, within the M%.

The data obtained from the vital alkaline phosphatase (ALP) staining are shown in **Figure 3** and **Supplementary Figure S3**. The effect of phosphate as factor was not significant overall, but with specific fungal patterns between the two *R. irregulare* Pi phenotypes. The F%, M%, m% and A% values were significantly higher under high Pi than under low Pi for the RiQS81-Pi^-^. For the RiQS81-Pi^+^ fungi, the M%, m% and A% values were significantly lower under high Pi than under low Pi. The impact of Pi phenotypes was significant with two contrasting situations. Under low Pi, inoculation with RiQS81-Pi^+^ inoculum was associated with significantly higher colonization for all fungal parameters assessed. Under high Pi, RiQS81-Pi^+^ inoculation (i) led to a significantly higher F% value, and (ii) has no significant influence for all other fungal parameters investigated. Furthermore, we noticed that vesicles were dark stained under high Pi but not, or less so under low Pi (**Supplementary Figure S4**), and their abundance was significantly higher within the Pi^+^ phenotype under low Pi conditions. Finally, the vesicle abundance increased significantly with increasing Pi concentration for the Pi^-^ phenotype, but not for the Pi^+^ one. The percentages of fungal structure stained by ALP within the ink-stained part (**Supplementary Figure S5**) showed similar profiles for all fungal parameters except for F%: the highest values were obtained under high Pi RiQS81-Pi^-^ (strongly and significantly higher than under low Pi), while the values obtained from RiQS81- Pi^+^ were intermediate and without significant differences between low and high Pi. Significant differences were observed between RiQS81-Pi^-^ and RiQS81-Pi^+^, the latter being associated with significantly higher values under low Pi for F%, M% and v%, but significantly lower values under high Pi for M%, m%, V% and A%).

### 3.2 The adaption process modulated the transcription of fungal and plant genes

#### 3.2.1 Plant genes

**Figure 4:**
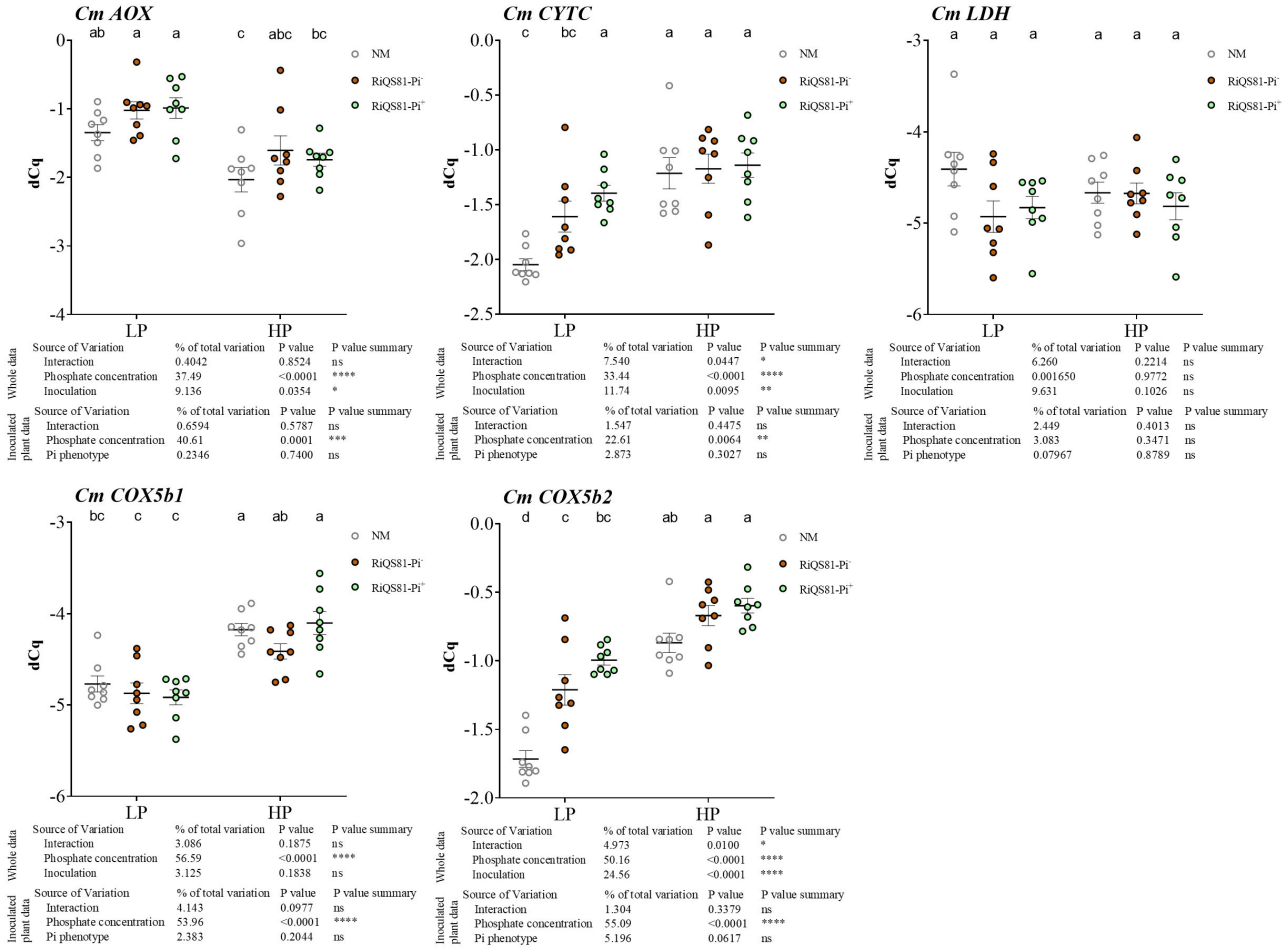
Expression patterns of melon genes involved in respiratory pathways and fermentation in mycorrhizal and non-mycorrhizal roots at different Pi fertilizer levels. Plants were inoculated with RiQS81-Pi^-^ or RiQS81-Pi^+^ inocula. Roots were harvested at 77 DAI, RNA extracted, and expression measured by qRT-PCR of the genes encoding alternative oxidase (AOX), cytochrome C (CytC), two isoforms of the subunit 5b of the cytochrome oxidase (COX5b) and lactate dehydrogenase (LDH). Genes encoding β-actin (ACT) and ADP-ribosylation factor-1 (ADP) were used for normalization. Data show mean expression level (*n* = 8) ± s.e. Treatments sharing the same letter are not significantly different (*p* < 0.05; Tukey multiple-comparison ANOVA 2 ways, with assessment on the whole design and when considering inoculated plants alone). The tables provide statistical details per factor; significant differences are indicated by stars (ns: non-significant; * P < 0.05; ** P < 0.005;*** P < 0.0005; **** P < 0.00005). NM, Non-inoculated plants; HP, high phosphate; LP, low phosphate.

The transcript levels of melon genes involved in respiratory and fermentative pathways were investigated (**Figure 4**). The gene encoding the melon alternative oxidase *CmAOX* was repressed by Pi, while the genes involved in the COX pathway (*CmCOX5b1*, *CmCOX5b2* and *CmCytC*) were induced by Pi. *CmCytC* and *CmCOX5b2* showed a similar expression profile, with a higher expression in the inoculated samples under low Pi, as well as a higher transcription level in the plants inoculated with the RiQS81-Pi^+^ strain under this condition. The fungal Pi phenotype status of the inoculum played no role in the transcript accumulation of these genes in high Pi conditions. The lactate dehydrogenase *CmLDH* was constitutively expressed in our experimental conditions.

**Figure 5:**
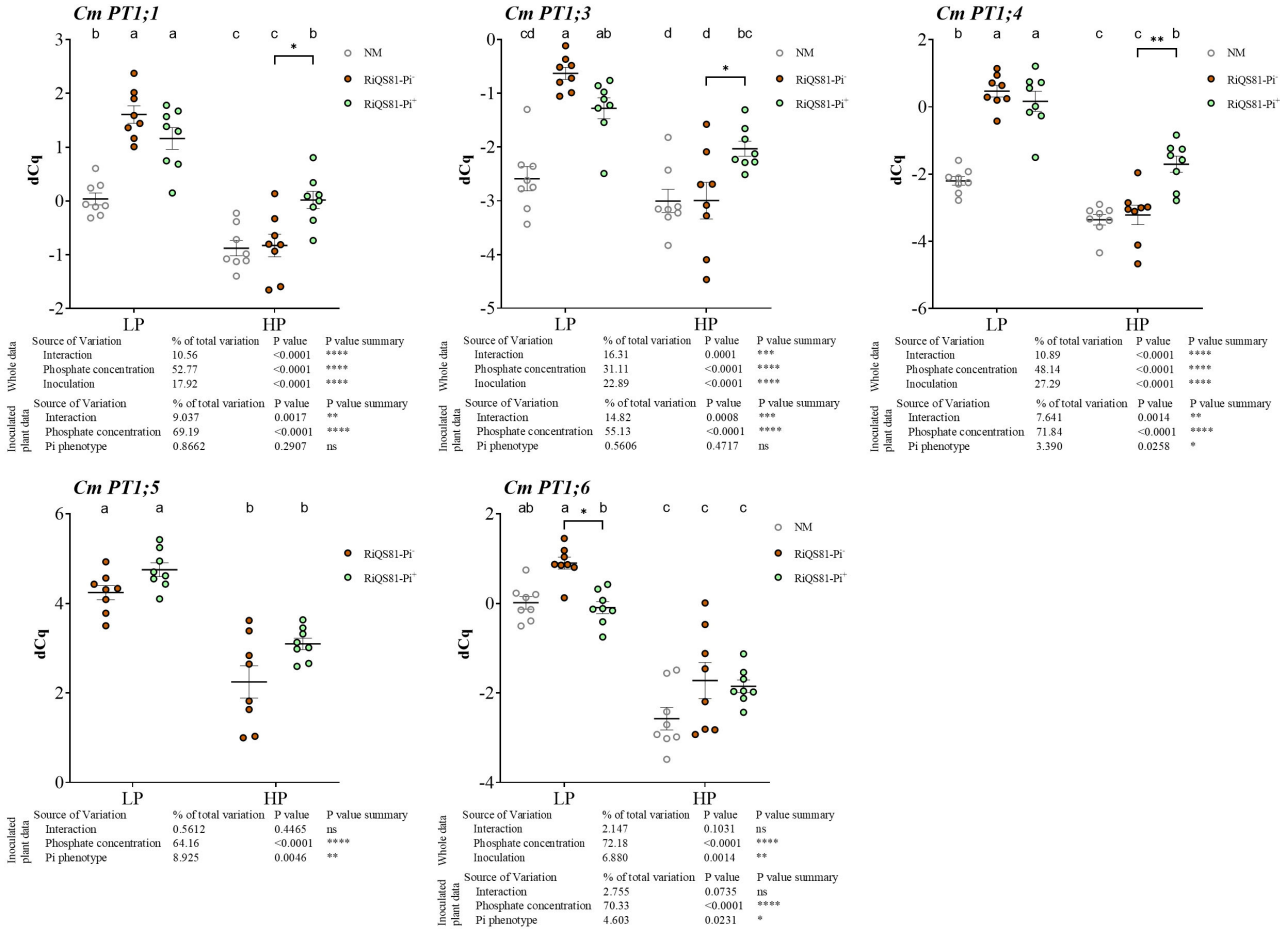
Expression patterns of melon Pi transporter genes belonging to the PHT1 family in mycorrhizal and non-mycorrhizal roots at Pi fertilizer levels. Plants were inoculated with RiQS81-Pi^-^ or RiQS81-Pi^+^ inocula. Roots were harvested at 77 DAI, RNA extracted, and expression measured by qRT-PCR of the genes encoding the transporters CmPT1;1, CmPT1;3, CmPT1;4, CmPT1;5 and CmPT1;7. Genes encoding β-actin (ACT) and ADP-ribosylation factor-1 (ADP) were used for normalization. Data show mean expression level (*n* = 8) ± s.e. Treatments sharing the same letter are not significantly different (*p* < 0.05; Tukey multiple-comparison ANOVA 2 ways, with assessment on the whole design and when considering inoculated plants alone). The tables provide statistical details per factor; significant differences are indicated by stars (ns, non-significant; * P < 0.05; ** P < 0.005;*** P < 0.0005; **** P < 0.00005). Note that for Cm PT1;5, the non-inoculated plants data are not represented due to inexistent expression. NM, Non-inoculated plants; HP, high phosphate; LP, low phosphate.

Based on the genome sequence of the melon, we identified seven Pi transporter genes belonging to the *Pht1* family in melon, designated *CmPT1;1* to *CmPT1;7*, respectively. Phylogenetic analysis revealed that the *CmPT1;5* gene clustered with the AM-specific Pi transporters previously characterized in numerous plant species (**Supplementary Figure S6**). The expression levels of Pht1 genes were studied in melon roots under high or low Pi availability, either in the absence of AMF or colonized by RiQS81-Pi^-^ or RiQS81-Pi^+^ *R. irregulare* (**Figure 5**; **Supplementary Figure S7**). *CmPT1;2* and *CmPT1;7* were very weakly expressed in the root samples, but strongly expressed in other parts of the plant (**Supplementary Figure S8**) and were not further considered in the analysis. All other Pi transporters were repressed by high Pi and most of them were significantly upregulated by inoculation, except *CmPT1;6*. *CmPT1;5* was strongly upregulated in the presence of AMF, as expected from its similarity to previously characterized AMF-inducible Pi transporter genes. *CmPT1;1*, *CmPT1;3* and *CmPT1;4* showed a similar profile: in LP conditions, all three were induced by AMF, and in HP, the repression by the Pi was less strong in the presence of the RiQS81-Pi^+^ strain. Similar to all known AMF-inducible Pi transporters, the expression levels were correlated with the mycorrhizal colonization of the roots, suggesting that the difference between RiQS81-Pi^+^ and RiQS81-Pi^-^ is most likely due to the different colonization of the roots.

**Figure 6:**
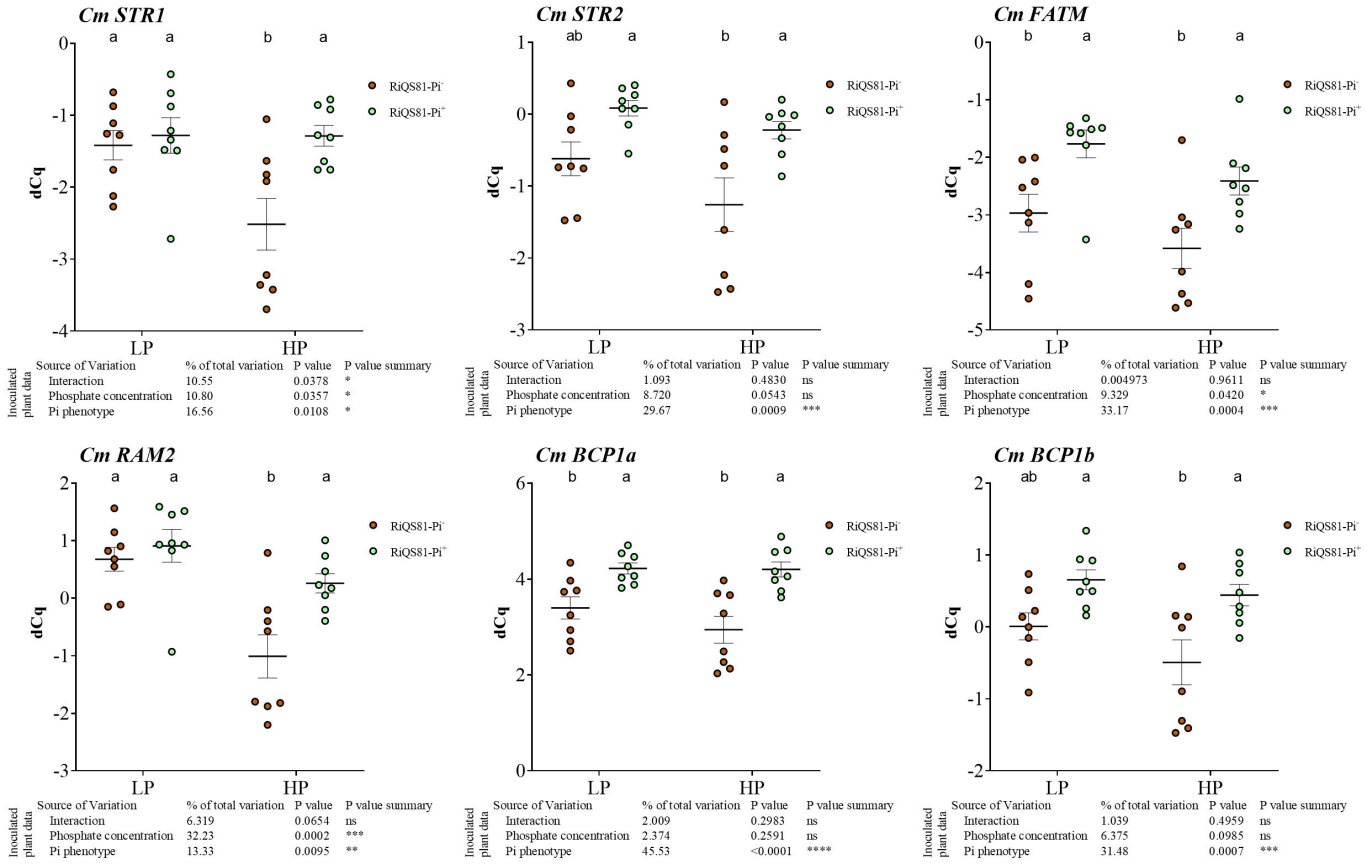
Expression patterns of melon genes involved in fatty acid biosynthesis and transport, and blue copper protein genes in mycorrhizal and non-mycorrhizal roots at Pi fertilizer levels. Plants were inoculated with RiQS81-Pi^-^ or RiQS81-Pi^+^ inocula. Roots were harvested at 77 DAI, RNA extracted, and expression measured by qRT-PCR of the genes encoding two half size ATP-binding cassette transporters of subfamily ABCG (STR1 and STR2), a thioesterase (FatM), a glycerol-3-P acyltransferase (RAM2), and two Blue Copper-Binding Proteins (BCP1a and βCP1b). Genes encoding β-actin (ACT) and ADP-ribosylation factor-1 (ADP) were used for normalization. Data show mean expression level (*n* = 8) ± s.e. Treatments sharing the same letter are not significantly different (*p* < 0.05; Tukey multiple-comparison ANOVA 2 ways, with assessment on the whole design and when considering inoculated plants alone). The tables provide statistical details per factor; significant differences are indicated by stars (ns, non-significant; * P < 0.05; ** P < 0.005; *** P < 0.0005; **** P < 0.00005). NM, Non-inoculated plants; HP, high phosphate; LP, low phosphate.

Melon orthologs of genes involved in fatty acid biosynthesis and transport previously described in other species were identified and named as follows: *CmFatM* (XM_008468942.3) encoding a thioesterase; *CmRAM2* (XM_008445223.3) encoding a glycerol-3-P acyltransferase; *CmSTR1* (XM_008447787.3) and *CmSTR2* (XM_051088815.1) encoding two half size ATP-binding cassette transporters of subfamily ABCG (**Supplementary Figures S9**, **S10**). All these genes were strongly upregulated by the presence of AM fungi in roots. In mycorrhizal plants, they were all upregulated in RiQS81-Pi^+^ under both low and high Pi conditions (**Figure 6**). For *CmSTR1* and *CmSTR2*, the fungal Pi^+^ phenotype allowed to restore the same induction level in HP as in LP, although the mycorrhizal colonization was lower.

Certain Blue Copper-Binding Proteins (BCP) were previously identified as being encoded by AMF-inducible genes (Parádi et al., 2010). Their role remains unknown, but melon, like *Medicago*, possesses several AMF-specific BCPs. In this study, *CmBCP1a* (XM_008438947.3) and *CmBCP1b* (XM_008439498.1) were identified and investigated. Both were strongly induced by AMF, and as for the genes involved in fatty acid metabolism, the fungal Pi^+^ phenotype restored the induction levels of LP in HP (**Figure 6**).

#### 3.2.2 Fungal genes

As for the host plant, fungal genes involved in respiration and nutrient uptake were studied, as well as some previously described genes involved in the Pi signaling pathway.

**Figure 7:**
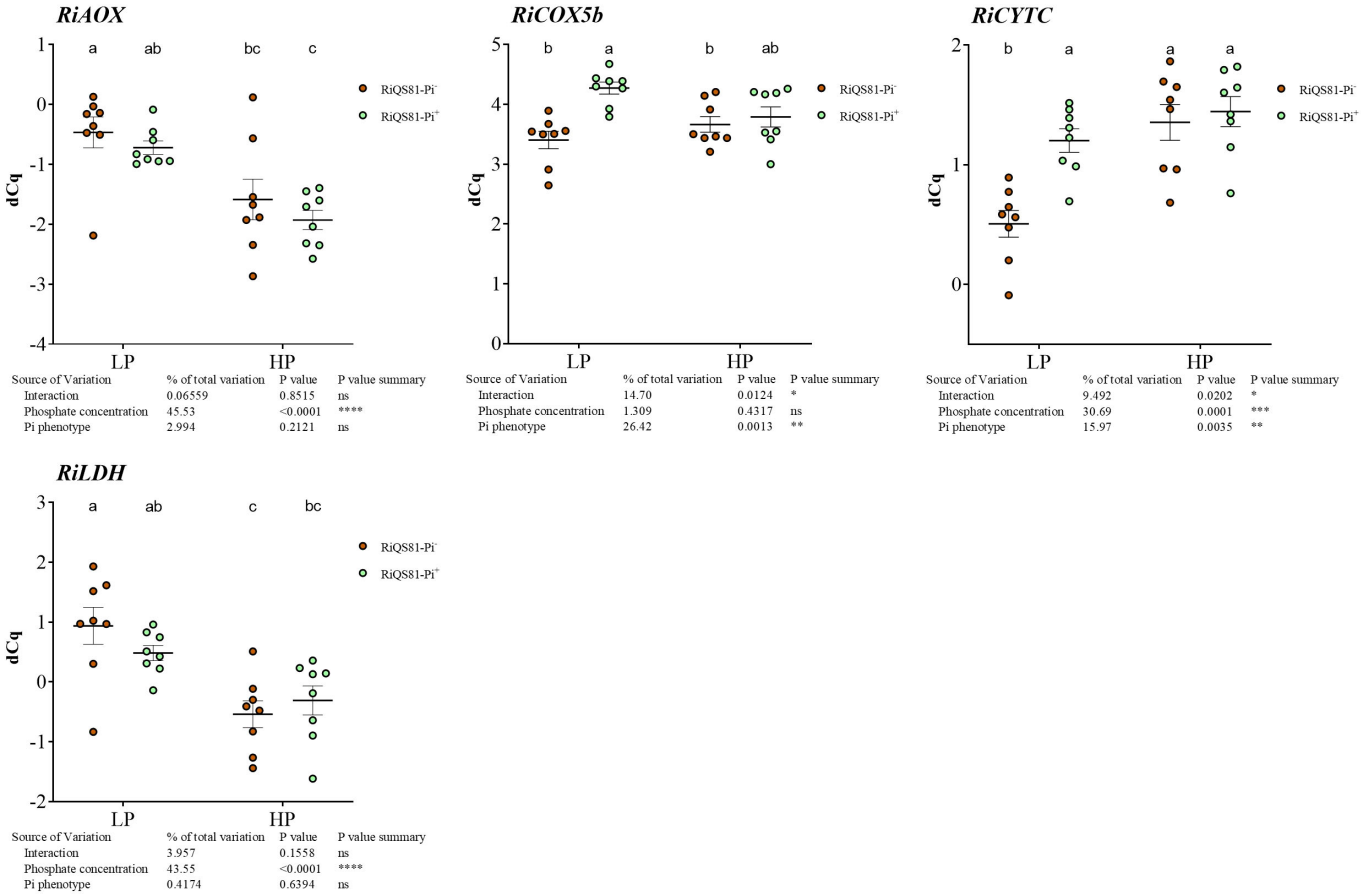
Expression patterns of R. irregulare genes involved in respiratory pathways and fermentation in mycorrhizal melon roots at different Pi fertilization levels. Plants were inoculated with RiQS81-Pi^-^ or RiQS81-Pi^+^ inocula. Roots were harvested at 77 DAI, RNA extracted, and expression measured by qRT-PCR of the genes encoding alternative oxidase (AOX), cytochrome C (CytC), the subunit 5b of the cytochrome oxidase (COX5b) and lactate dehydrogenase (LDH). Gene encoding isocitrate lyase (ICL) was used for normalization. Data show mean expression level (*n* = 8) ± s.e. (*p* < 0.05 Tukey multiple-comparison ANOVA 2 ways, with assessment on the whole design and when considering inoculated plants alone). The tables provide statistical details per factor; significant differences are indicated by stars (ns, non-significant; * P < 0.05; ** P < 0.005; *** P < 0.0005; **** P < 0.00005). HP, high phosphate; LP, low phosphate.

Genes encoding the alternative oxidase, *RiAOX*, and the lactate deshydrogenase gene, *RiLDH*, were both down regulated by high Pi fertilization, but no impact of the *R. irregulare* Pi phenotype was observed (**Figure 7**). For the COX pathway, *RiCytC* was induced by Pi for the RiQS81-Pi^-^ fungus, but also by the fungal Pi phenotype under LP conditions. The expression of the sugar transporter gene *RiMST* showed a slight induction in the LP RiQS81-Pi^+^ condition, but the expression was mainly constitutive.

**Figure 8:**
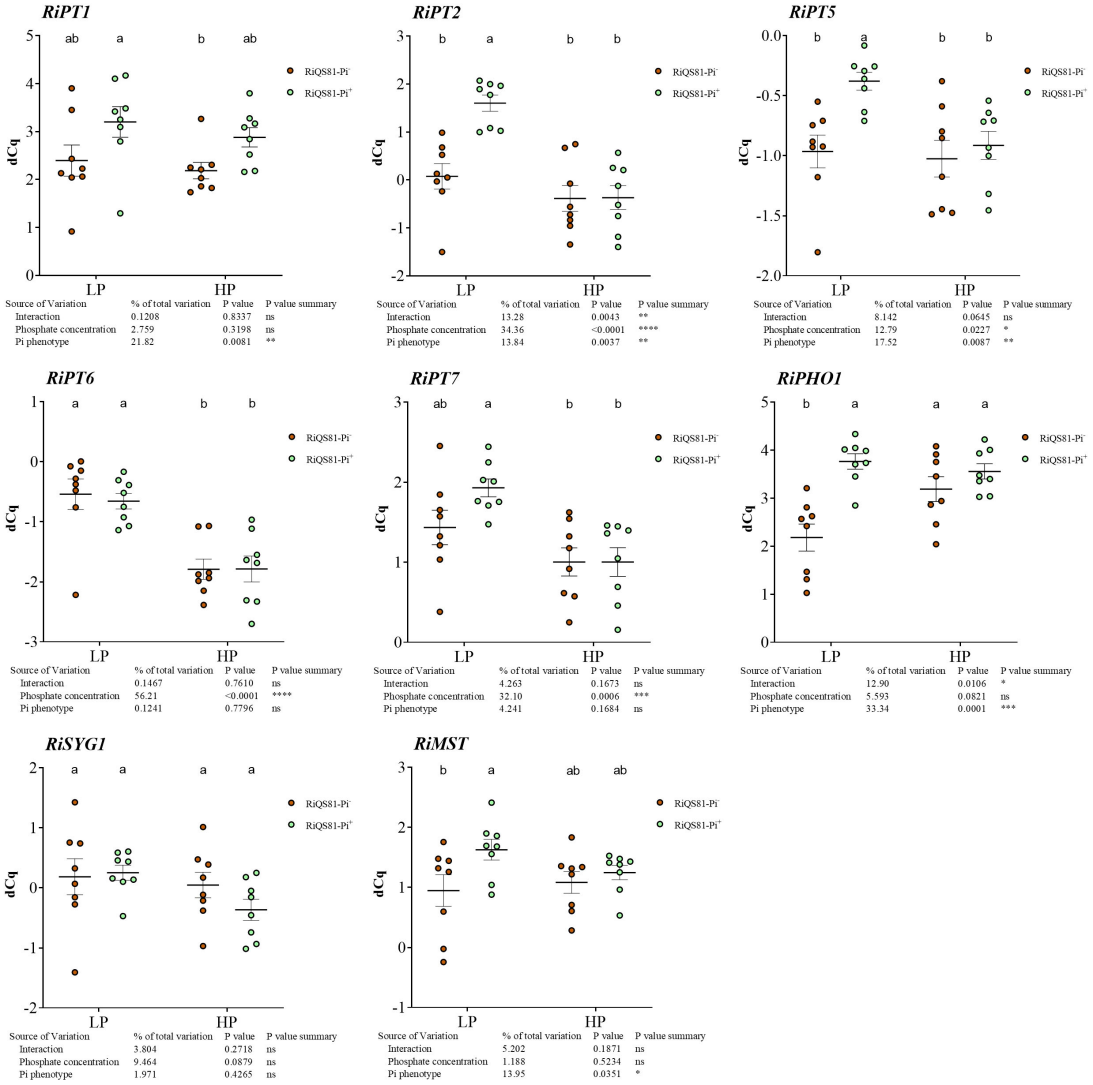
Expression patterns of R. irregulare Pi transporter genes belonging to the Pht1 family, and the sugar transporter gene RiMST in mycorrhizal melon roots at different Pi fertilization levels. Plants were inoculated with RiQS81-Pi^-^ or RiQS81-Pi^+^ inocula. Roots were harvested at 77 DAI, RNA extracted, and expression measured by qRT-PCR of the genes encoding phosphate transporters (PT1, PT2, PT5, PT6, PT7, PHO1, SYG1) and the sugar transporter MST. Gene encoding isocitrate lyase (ICL) was used for normalization. Data show mean expression level (*n* = 8) ± s.e. (*p* < 0.05 Tukey multiple-comparison ANOVA 2 ways, with assessment on the whole design and when considering inoculated plants alone). The tables provide statistical details per factor; significant differences are indicated by stars (ns, non-significant; * P < 0.05; ** P < 0.005;*** P < 0.0005; **** P < 0.00005). HP, high phosphate; LP, low phosphate.

To determine the role of fungal Pi transporters in the mycorrhizal association between Pi^-^ and Pi^+^ phenotypes, the expression of nine previously described fungal Pi transporters (Walder et al., 2016; Xie et al., 2022) was investigated in melon roots (**Figure 8**; **Supplementary Figure S11**). *RiPT3* and *RiPT4* presented a too high sequence similarity to design specific primers, and the expression of these two genes was very low to undetectable in our conditions; therefore, they were not considered for further analysis. *RiPT1* and *RiPHO1* were the most expressed Pi transporter genes within all conditions (**Supplementary Figure S11**). The transcript levels of *RiPT1* and *RiSYG1* were not affected by Pi fertilization or the *R. irregulare* phenotype. *RiPT6* and *RiPT7* were only affected by the Pi level and were both repressed in HP conditions. *RiPT2* and *RiPT5* presented a similar profile, with a higher expression level only in the LP RiQS81-Pi^+^ condition. *RiPHO1* was repressed in the LP RiQS81-Pi^-^ condition.

**Figure 9:**
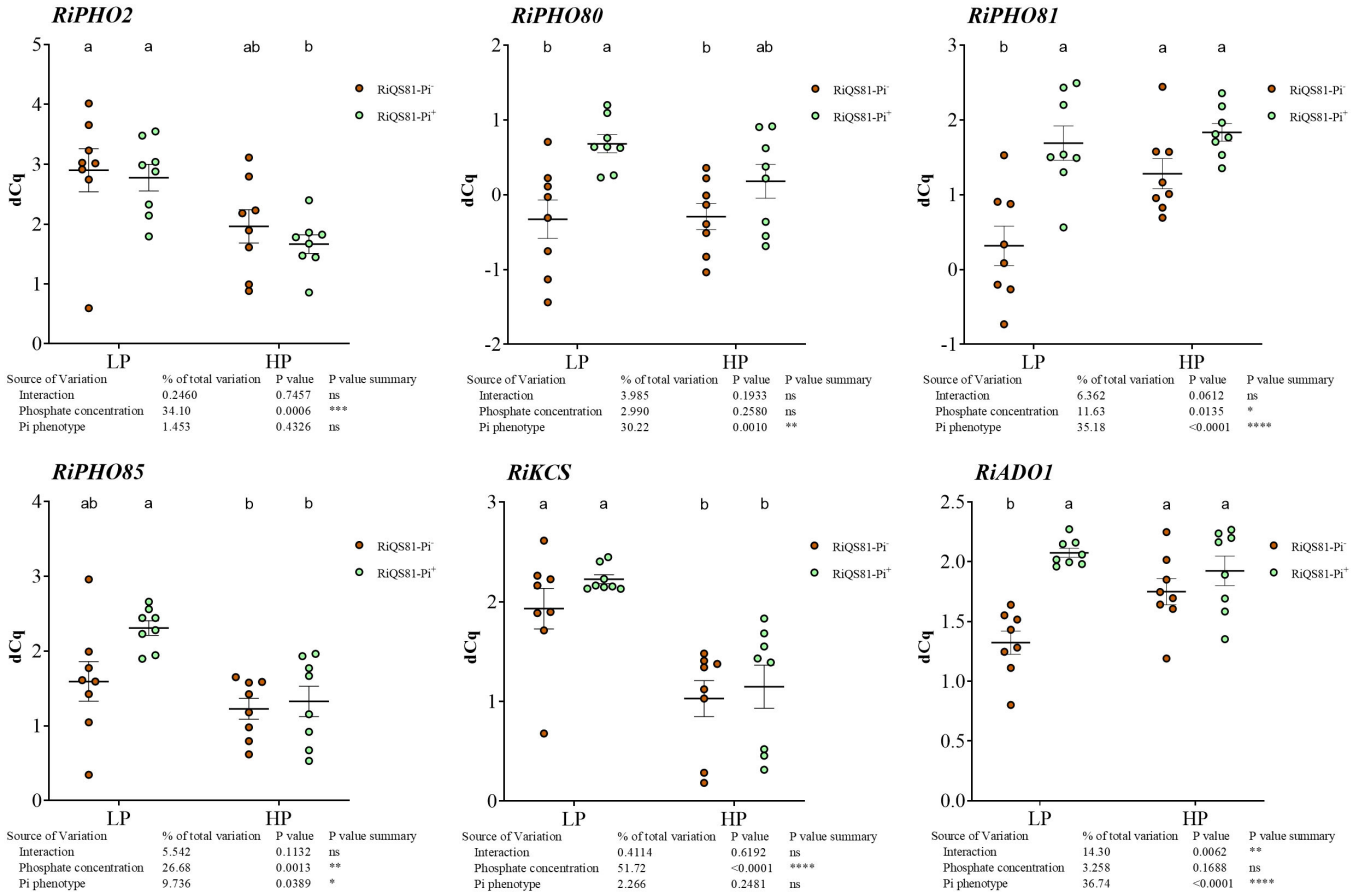
Expression patterns of R. irregulare genes involved in Pi-signaling in mycorrhizal melon roots at different Pi fertilization levels. Plants were inoculated with RiQS81-Pi^-^ or RiQS81-Pi^+^ inocula. Roots were harvested at 77 DAI, RNA extracted, and expression measured by qRT-PCR of the genes encoding adenosine kinase (ADO1), inositol hexakiphospate kinase (KCS1) and regulators of the PHO pathway (PHO2, PHO80, PHO81, PHO85). Gene encoding isocitrate lyase (ICL) was used for normalization. Data show mean expression level (*n* = 8) ± s.e. (*p* < 0.05 Tukey multiple-comparison ANOVA 2 ways, with assessment on the whole design and when considering inoculated plants alone). The tables provide statistical details per factor; significant differences are indicated by stars (ns, non-significant; * P < 0.05; ** P < 0.005;*** P < 0.0005; **** P < 0.00005). HP, high phosphate; LP, low phosphate.

To investigate the effect of external phosphate concentrations and fungal Pi phenotype on the transcript levels of AM fungal genes involved in Pi uptake and metabolism, the expression profiles of six genes of the PHO pathway (Zhou et al., 2021; Xie et al., 2022) of *R. irregulare* in mycorrhizal *C. melo* roots were assessed (**Figure 9**). Our data show (i) a response to high Pi in Pi^-^ phenotype in *RiKCS1* (down-regulation), *RiADO1*, *RiPHO81* (up-regulation), (ii) a response to high Pi in Pi^+^ phenotype in *RiPHO2, RiPHO85* and *RiKCS* (down-regulation) and (iii) significant differences between the two *R. irregulare* phenotypes for *RiPHO80, RiPHO81*, and *RiADO1* occurring only under low Pi conditions.

In conclusion, for each of the partners of the symbiosis, the Pi adaptation process influenced the accumulation of specific transcripts. In the fungus, we can distinguish between genes that respond only to Pi, those that are upregulated in RiQS81-Pi^+^ in low Pi and those whose expression is lower only in RiQS81-Pi^-^ in low Pi (**Supplementary Figure S12A**). Similarly, in the plant, the expression data can be clustered into genes responding only to Pi, those that respond to inoculation with the expression following the colonization rate, and those for which inoculation with the Pi+ phenotype restored an expression similar to LP in HP, regardless of the mycorrhization rate (**Supplementary Figure S12B**). Several significant correlations between phenotypic patterns and transcriptomic patterns were also obtained (**Supplementary Figures S13**-**S16**).

### 3.3 Continuous *ex vitro* inoculum production under high P led to higher propagule production

**Figure 10:**
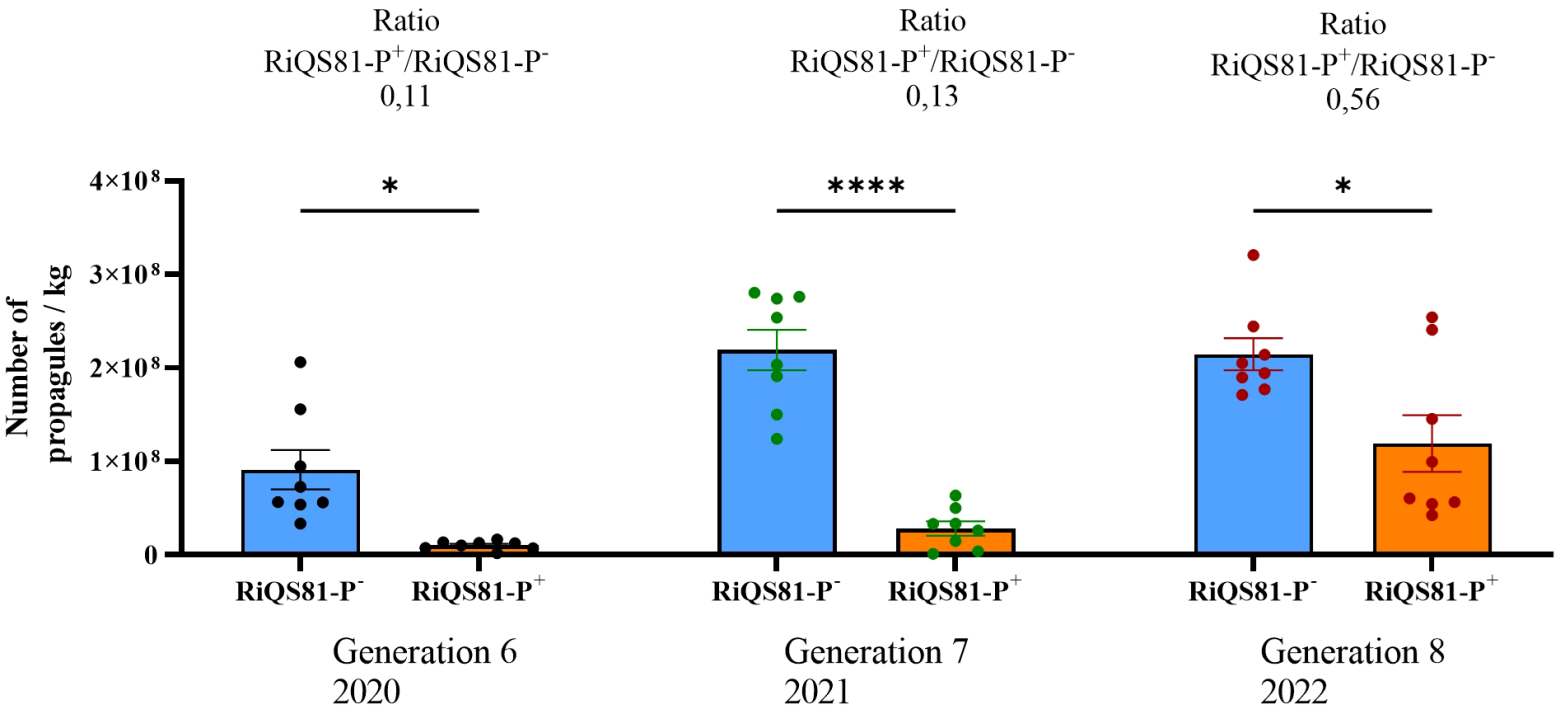
Number of propagules per kilo for RiQS81-Pi^-^ and RiQS81-Pi^+^ inocula produced under *ex vitro* condition (*greenhouse*). Generation 6: produced in 2020, inoculation with spores from the 5th *in vitro* generation; generation 7: produced in 2021, inoculation with powder inoculum from 2020; generation 8: 2022, inoculation with powder inoculum from 2021. Data show mean expression level (*n* = 8) ± s.e. Significant differences are symbolized by stars (* P < 0.05; **** P < 0.00005; Tukey multiple-comparison ANOVA 2 ways, significant differences are represented only between RiQS81-Pi^-^ and RiQS81-Pi^+^ for each production year). RiQS81-Pi^-^: *R. irregulare* produced in presence of 10 ppm phosphorus inside the substrate; RiQS81-Pi^+^: *R. irregulare* produced in presence of 100 ppm phosphorus inside the substrate.

In parallel to the melon pot trial, the production of *R. irregulare* under high Pi was pursued *ex vitro* following the same protocol as for the 6^th^ generation (10 ppm Pi to produce the RiQS81-Pi^-^, and 100 ppm Pi to produce RiQS81-Pi^+^, using *Plantago lanceolata*). While Pi had a strong effect on reducing fungal propagules in the 6th generation, this effect tended to be buffered after 2 more generations (7th and 8th generation in 2021 and 2022, respectively), with an increased ratio of propagules between RiQS81-Pi and RiQS81-Pi^+^ production (**Figure 10**).

## 4 Discussion

### 4.1 RiQS81-Pi^-^ and RiQS81-Pi^+^ differ in fungal phenotype and functionality within melon roots

We hypothesized that *R. irregulare* can be artificially adapted to exhibit increased tolerance to high phosphate concentrations, and that this adaptation process is accompanied by phenotypic changes in both the fungus and the host plant. Our data strongly support these hypotheses, as the adaptation conferred a new functionality with positive effects on plants under low Pi conditions and a partial alleviation of the negative MGR response typically observed under high Pi (Wang et al., 2016). Endomycorrhizal symbiosis involves a cost/benefit balance (Wipf et al., 2019), where the plant expends energy that the fungus uses to produce propagules (spores, vesicles) and mycelium, while the fungus exchanges nutrients through arbuscules that benefit plant growth. Consistent with this perspective, our results support the notion that the occurrence of the arbuscule/vesicle ratio partially modulates the plant response. Vesicles are thick-walled lipid-storing organs (Jabaji-Hare et al., 1984; Brundrett, 1991), and it is reasonable to assume that their abundance reflects the fungal energy status (resilience and propagule fitness), which may influence the benefits to the plant. Interestingly, our data demonstrate that compared to RiQS81-Pi^-^, the abundance of vesicles in RiQS81-Pi^+^ is reduced under low Pi conditions but increased under high Pi conditions, indicating a differential fitness between the two fungal phenotypes in response to Pi concentrations. This suggests that the RiQS81-Pi^+^ phenotype is associated with a shift in the Pi tolerance range. Furthermore, this shift implies that Pi concentration induces distinct stresses in the fungus: RiQS81-Pi^-^ is sensitive to Pi excess, whereas RiQS81-Pi^+^ is sensitive to what could be perceived as Pi starvation (Huo et al., 2022). This observation suggests that the Pi phenotype is linked to changes in Pi sensing, uptake, and metabolism, which are important characteristics for distinguishing adaptation from acclimatization.

The histochemical staining of alkaline phosphatase is considered to be a marker of polyphosphate metabolic activity localized in fungal tissues within the roots, and usually coincides with plant growth stimulation. It is also known that the intensity of alkaline phosphatase decreases with the age of colonization or when soluble phosphate is added to the substrate, and that this metabolic activity is associated with mycorrhizal functionality and ability to transfer phosphate to the plant (Tisserant et al., 1993; Vivas et al., 2003; Aono et al., 2004; Funamoto et al., 2007; Zhang et al., 2023). Our data are partially consistent with these previous observations when comparing both fungal phenotypes under low Pi. However, surprisingly, the proportion of fungal structures stained by ALP under high Pi increased with RiQS81-Pi^-^ and remained unchanged with RiQS81-Pi^+^, but was associated with a moderately significant correlation with plant biomass and shoot fresh weight. Interestingly, ALP staining was observed in arbuscules, intraradical hyphae, but also within vesicles, indicating the possible occurrence of active mitochondrial processes where energy is required and where polyphosphate metabolism is involved. A much smaller proportion of vesicles were stained by ALP compared to ink in the LP RiQS81-Pi condition, but the presence of intact lipid bodies would suggest that the vesicles are still alive but have probably entered a dormant, i.e. late stage. This would suggest that ALP staining is a marker of specific phosphate metabolic activity associated with physiological age.

Of note, the culture system used in this study consisted of relatively small pots filled with an inert substrate and a fertilizer containing only readily available forms of nutrients. Apart from the effect of Pi concentration, such a system can result in a neutral or occasionally negative MGR (Malcová et al., 2001; Audet and Charest, 2010; Zangaro et al., 2015; Qin et al., 2022), which was the case for RiQS81-Pi^-^ under low Pi. However, we also observed higher transcription of the AM-inducible Pi transporter as well as higher ALP activity under this condition. This is consistent with previous work concluding that a negative or neutral MGR does not necessarily indicate a high carbon cost to the plant or a reduced supply of phosphate nutrients from the fungus to the plant (Smith et al., 2003, 2004; Nouri et al., 2014). This supports the idea that the MGR is intricately linked to the physiological state of the plant and involves complex metabolic components. Overall, we observed distinct fungal phenotypic profiles for each of the four MGR statuses obtained, suggesting that the MGR corresponds to a function of mycorrhizal activity that is unrelated to fungal fitness. Therefore, when interpreting the adaptation traits, it is important to primarily consider and compare the fungal parameters between the two inoculation conditions at a given Pi concentration. In this respect, RiQS81-Pi^+^ promotes MGR as compared with RiQS81-Pi^-^, suggesting that the adapted *R. irregulare* may have an alternative Pi metabolism, possibly related to nutrient transfer.

As remark, Smith and Smith (2011) pointed out that the colonization rate would not be a relevant measure of fungal biomass, as it should also consider root length, which is affected by Pi concentration. For this reason, we emphasize the importance of considering the relative abundance of interconnected mycorrhizal structures, rather than each of them independently as classically assessed by the original Trouvelot method. The relative proportions of arbuscule, vesicle and intraradical mycelium, as well as the proportion of ALP stained within the ink, are probably better phenotypic markers of the physiological status of the fungus.

### 4.2 Influence of the adaptation process on transcriptional responses in plants and fungi

#### 4.2.1 Fungal nutrient transport and Pi signaling

PT1 is the most studied Pi transporter in AMF. Its regulation by Pi remains uncertain, with conflicting results. Xie et al. (2016) and Zhang et al. (2023) reported that *PT1* was repressed by Pi in *Gigapora margarita* and *R. irregulare*, whereas Fiorilli et al. (2013) found no Pi regulation of this gene in *R. irregulare*. In our study, *RiPT1* transcript levels were not affected by Pi, but a significant and positive response to Pi adaptation was observed. RiPT1 has been proposed to be mainly responsible for the Pi uptake by the extraradical mycelium (Xie et al., 2022). However, the strong and constitutive expression of *RiPT1* independent of the Pi concentration in our study highlights the importance of this transporter also at the root level.

The available data on other AMF PTs are limited, sometimes contradictory, and their role in the symbiosis remains to be defined. *RiPT5* was shown by Walder et al. (2016) as the only Pi transporter gene in their study, which presented a trend of correlation between mycorrhizal Pi acquisition in plants and its level, defining it as the most promising candidate involved in intraradical Pi transfer to PAS. In more recent work, PT6, as PT1, was proposed to be mainly responsible for Pi uptake by the ERM, while the SPX domain-containing transporters, such as PT7 and PHO1, may be involved in Pi export at the symbiotic interface (Ezawa and Saito, 2018; Xie et al., 2022). The transcription of *PT7* was shown to be Pi concentration-independent, but necessary for arbuscule development and symbiotic Pi delivery under medium to low Pi concentrations. Indeed, inactivation of this gene resulted in premature arbuscule degradation, and RiPT7 was hypothesized to play an important role in *R. irregulare* development within roots at late stage, with Pi delivered by this transporter acting as a signal for continued AMF symbiont development in roots (Xie et al., 2022). In our study, *RiPT6* was the most strongly correlated with ink fungal parameters, although it was only regulated by Pi levels and was repressed by HP. Interestingly, transcripts of *RiPHO1*, *RiCOX*, *RiMST* and several fungal ALP parameters (especially the relative arbuscule abundance) are all positively and significantly correlated, suggesting a higher metabolic and exchange activity in the Pi^+^ phenotype. The sugar transporter gene *RiMST2* has been shown to be expressed in the IRM and its down-regulation resulted in reduced root colonization (Helber et al., 2011), suggesting a role in sugar translocation from the plant to the fungus. These observations under our experimental conditions would be more consistent with a role for RiPHO1 in the exchange between the host and the fungal partner.

*RiKCS1, RiADO1, RiPHO80, RiPHO81, RiPHO85 and RiPHO2* were selected to gain insight into the possible modulation of the fungal PHO pathway between the adapted and non-adapted fungus. Few data are available on these genes in AMF. In *Gigaspora margarita, pho81* transcripts were induced under low Pi and repressed under high Pi, whereas the expression of *pho80* and *pho85* was not affected by ambient Pi levels, suggesting a primary sensing by Pho81 whose expression levels are largely responsive to external Pi levels (Xie et al., 2016). Our data show a different pattern of gene regulation, partially consistent with the Pi response, with differences between the two fungal Pi phenotypes. *RiPHO2, RiPHO85, and RiKCS1* were the only P-responsive genes, whereas *RiADO1, RiPHO81*, and *RiPHO80* responded to the Pi adaptation process under LP conditions. These observations again question the physiological status of the LP RiQS81-Pi^-^ plants and point to possibly less active fungal structures in this condition or a modulation of Pi sensing in the RiQS81-Pi^+^ strain, linked to a higher capacity to colonize the plants.

#### 4.2.2 Plant P transport

A number of studies have shown that AM colonization of plants down-regulates the expression of the DPU Pi transporters (Rausch et al., 2001; Burleigh, 2002; Glassop et al., 2005; Requena, 2005; Nagy et al., 2006) while other studies demonstrated different results. Chen et al. (2007) showed a mycorrhiza-induced down-regulation of *Pht1;1* and *Pht1;2* expression under low-Pi conditions, but Pi-regulation of *Pht1;2* under high-Pi conditions for pepper, eggplant, and tobacco. In tomato, Nagy et al. (2009) found no change in the transcript abundance for *SlPT1* and *SlPT2* upon root colonization. Our data are consistent with these latter observations, as presence of AMF did not induce downregulation of melon Pht1 transporters in any of our conditions. All melon Pht1 transporters expressed in the roots were downregulated by Pi, and all except *CmPT1;6* were significantly upregulated by the presence of AMF, with *CmPT1;5* expressed exclusively in AM roots. *CmPT1;1*, *CmPT1;3, CmPT1;4* and *CmPT1;5* were up-regulated in plants inoculated with RiQS81-Pi^+^ in HP conditions, in direct correlation with the mycorrhizal colonization parameters (**Supplementary Figure S15**).

#### 4.2.3 Respiration

Although one cannot necessarily deduce the enzymatic activities from RNA accumulation for the genes involved in respiratory pathways (Sluse and Jarmuszkiewicz, 1998), the results show, both in the plant and the fungus, a down regulation of genes involved in the AOX pathway associated with an upregulation of genes involved in the COX pathway in high Pi conditions, which was expected (Bedini et al., 2018). Consistently, strong correlations were obtained between the mitochondrial respiration pathway components with plant biomass (**Supplementary Figures S12**, **S13**). In agreement with previous reports (Del-Saz et al., 2017), upregulation of plant genes involved in the COX pathway (*CmCytC* and *CmCOX5b2*) were induced by the presence of AMF in low Pi conditions, with prominent response with the Pi^+^ phenotype. This upregulation is reliable with positive MGR obtained with RiQS81-Pi^+^, but not with the negative MGR observed with RiQS81-Pi^-^. This would be coherent with Smith and Smith (2011), who argued that a negative MGR does not translate to a parasitic fungal behavior since a transfer of Pi and carbon still occur.

In the fungus, significant differences were also observed between the two Pi phenotypes, mainly under low Pi condition (*RiCOX5b* and *RiCytC*). This could indicate, in relation with the ALP straining results, a higher metabolic activity of Pi^+^ phenotype. We observed a positive correlation between the ALP stained fungal structures and the transcript abundance of fungal genes as *RiCOX*, *RiMST* and *RiPHO1* (**Supplementary Figure S12**), reinforcing the hypothesis that the ALP activity could be related with fungal metabolic process linking sugar uptake, Pi transport and mitochondrial activity (Kizawa et al., 2016; Ortíz et al., 2020). A link between ALP parameters and *RiCOX* transcripts makes sense, since it was reported that the inhibition of the COX pathway by KCN suppress also alkaline phosphatase activity (Van Belle, 1972; Ezawa et al., 1995). On the other side, plant parameters are positively correlated with *RiCytC* transcripts. This would be consistent with a nutrient delivery from fungus to plant, as an active process requiring ATP provided by the COX pathway. In conclusion, mitochondria are major actors in mycorrhizal association and play a role in fungal tolerance to Pi.

It was previously hypothesized that the LDH could possibly be related to AOX and fermentation in AMF, to sustain energy demand (Bedini et al., 2018). This could be partially comforted by our results where the expressions patterns of *RiAOX* and *RiLDH* were similar, as well as *CmAOX* and *RiLDH*, but no correlation was found between *CmAOX* and *CmLDH.* Moreover, we noticed that both *RiAOX* and *RiLDH* were significantly and positively correlated with fungal ink parameters, and notably with the relative arbuscule and vesicle abundance, but negatively correlated with the proportion of ALP-stained structures within ink. It would therefore indicate that the fungal structures which were not stained by ALP were also viable, with a metabolism associated with the AOX pathway. This might translate to a state of dormancy notably for the vesicles. Therefore, the divergent proportion of ALP under low Pi condition between both *R. irregulare* phenotypes would be related to a differential physiological time, Pi^-^ being more advanced in the life stage than Pi^+^ and inversely under high Pi.

#### 4.2.4 Lipid metabolism and transport

Beyond phosphate transport and signaling, other genes related to symbiotic function have been investigated, such as those involved in fatty acid biosynthesis and transport. Arbuscular mycorrhizal fungi (AMF) lack the ability to synthesize fatty acids due to the absence of cytosolic type I fatty acid synthase genes, suggesting their reliance on the plant for fatty acid delivery. This dependence is further supported by the discovery of an AM-specific lipid biosynthetic pathway, and the upregulation of lipid biosynthesis and transporter genes in colonized cortical cells during symbiosis (Zhang et al., 2010; Gutjahr et al., 2012; Tisserant et al., 2013; Bravo et al., 2017; Jiang et al., 2017; Keymer et al., 2017; Luginbuehl et al., 2017; Morin et al., 2019; Malar C et al., 2021). The melon orthologs of the gene encoding the thioesterase FatM, the GPAT RAM2, and a pair of ABCG half-transporters unique to mycorrhizal plants and necessary to support arbuscule formation, named STR1 and STR2 (Zhang et al., 2010; Gutjahr et al., 2012; Bravo et al., 2017; Jiang et al., 2017), were examined. All of these AM-inducible plant genes, and particularly *CmRAM2*, were positively correlated with several fungal (ink) parameters, especially arbuscle abundance (**Supplementary Figure S12**). The transcript levels of some of these plant genes were significantly repressed by high Pi in the presence of the *R. irregulare* Pi^-^ phenotype, which could be consistent with lower colonization; however, this is not the case for the Pi^+^ phenotype. Remarkably, all these plant genes are significantly differentially regulated between the two *R. irregulare* phenotypes under high Pi condition, as well as *CmFATM* under low Pi condition.

While a link between fungal colonization (ALP), *RiMST* and *RiCOX* was observed, indicating a consistent flow from sugar uptake and assimilation from arbuscule to the mitochondria, the fate of lipid seems to correlate modestly to this metabolic path. It would be interesting to see in a future trial if a stronger correlation exists with the extraradical spore density.

#### 4.2.5 Blue copper proteins

Blue copper proteins (BCPs), characterized by their involvement in electron transfer reactions, have also been investigated in the context of AM roots. The AM-induced *BCP* family was shown as specifically and strongly Pi-regulated in arbuscule-containing regions of mycorrhizal roots, in the plasma membrane and the periarbuscular membrane around the arbuscule trunk (Pumplin and Harrison, 2009; Parádi et al., 2010). Although their precise role remains unclear, gene promoter activity studies suggest that BCPs may serve as mediators of electron transfer processes in arbuscule-containing cells and their vicinity (Hohnjec et al., 2005). We monitored the accumulation of transcripts encoding two melon orthologs of the mycorrhiza-responsive genes *MtBCP1* (Parádi et al., 2010). Similar to the transcript levels found for genes involved in lipid metabolism and transport, the expression patterns of *CmBCP1a* and *CmBCP2b* cannot be interpreted easily, but the significant and higher levels of transcript in RiQS81-Pi^+^ as compared to RiQS81-Pi^-^ is a clear result from the adaptation process.

Taken together, the molecular data suggests that the Pi tolerance status of the fungus had an influence on the molecular dialogue between the two partners of the symbiosis. It is interesting to note that most of the transcriptional differences between the two Pi phenotypes are observed under low Pi condition on the fungal side but are observed under high Pi on the plant side. We noticed fewer significant changes and larger standard errors for the fungal transcript dataset. One of the probable biases is that we used RNA extracted from whole mycorrhized roots, which means a mixture of different structures (vesicles, hyphae, arbuscules), each of which probably has different regulatory profiles. For a clearer answer about the exchanges with the plant, it would be necessary to perform the transcriptional analyses in arbuscocytes only. It would also be interesting to see how the genes related to signaling are regulated in the ERM, and in kinetics through the lifespan of the symbiosis.

### 4.3 Acclimatization or adaptation?

Micro-organisms, including AMF, are constantly evolving to thrive in specific environmental conditions and the complex genetics of AMF reflects their ability to adapt to a wide range of environmental variation. The genetic of AMF is complex due to the presence of multiple nuclei within a single isolate (Malbreil et al., 2014), and a high intra-isolate variation was observed at genome level, for rDNA as well as for protein-coding sequences (Corradi et al., 2007; Gibson and Reed, 2008; Savary et al., 2018). This variation was further confirmed also at transcript level, showing that the gene variants are indeed transcribed (Boon et al., 2010), and it was suggested that the different functional variants of a gene could be expressed in different environments (Oliveira et al., 2010).

In our research, we aimed to generate a directed selection of advantageous phenotypes of *R. irregulare* progeny with high Pi tolerance. This selection process refers to the ability of a genotype to produce different phenotypes induced by the changing environment (Lorant et al., 2020). Successful domestication of the *R. irregulare* QS81 isolate was achieved after several generations in which high Pi pressure was maintained. It is worth noting that the adaptation process resulted in distinct fungal phenotypes in high Pi, but also in low Pi conditions. Therefore, the terms ‘acclimatization’ or ‘acclimation’ are not relevant in this context, as they imply the reversible adjustment of phenotype in response to environmental conditions over a lifetime (Pinsky et al., 2013; Collier et al., 2018). We have observed phenotypic changes associated with transcriptomic variations indicating an adaptive response to high Pi (stressful) environments (Ghalambor et al., 2007). This suggests the presence of cryptic genetic variation, indicating the existence of genotypic and phenotypic variance in the *R. irregulare* isolate used in this study (Paaby and Rockman, 2014). Initial investigations on nuclear and mitochondrial rDNA sequences did not allow the identification of specific genetic variations associated with the induced high Pi^+^ phenotype. On the other hand, these methods covered only a very small part of the genome and may not be relevant for the differentiation of the adapted *R. irregulare*. Therefore, further in-depth work is needed to unravel the possible genetic mechanisms that contributed to the phenotypic variation, such as nuclear selection/filtration or epigenetic changes (Ehinger et al., 2012; Boon et al., 2013; Angelard et al., 2014; Kokkoris et al., 2020; Peña et al., 2020; Savary et al., 2020; Chaturvedi et al., 2021; Mojica and Kültz, 2022). This may allow the identification of markers that can discriminate between the two *R. irregulare* Pi phenotypes.

Based on our first results, we adopt the term ‘short-term adaptation’ to describe the high Pi tolerance observed in our study, following the definition proposed by (Brooks et al., 2011). Our work provides an initial proof of concept and raises further questions for future investigation: How many generations are required to observe the first phenotypic signs of high Pi tolerance? How many generations are required to achieve a positive mycorrhizal growth response under high Pi conditions after application of RiQS81Pi^+^?

### 4.4 Open questions and perspectives

#### 4.4.1 Do high Pi-tolerant AMF lines already exist in managed crop systems?

The possibility to induce *R. irregulare* Pi^+^ phenotype after a short number of generations raises the question of whether high Pi-tolerant AMF lines exist naturally. High Pi levels resulting from long-term high Pi fertilization are common in many conventional field soils (Cheng et al., 2013). The number of propagules in such soils may be low or even absent in some cases (Mäder et al., 2000; Oehl et al., 2003; Roy et al., 2017). However, other practices challenge the sole effect of Pi in inhibiting the mycorrhizal colonization and soil richness, such as tillage (Jansa et al., 2002; Castillo et al., 2006; Avio et al., 2013; Verzeaux et al., 2017), lack of cover crops (Soti et al., 2016), selection of low mycorrhizal plant species (Martín-Robles et al., 2018), or application of pesticides (Jin et al., 2013; Riedo et al., 2021; Edlinger et al., 2022). Moreover, a lack of Pi inhibition on the fungal community under field conditions has also been observed in long- or short-term fertilization approaches (Peña Venegas et al., 2021), but to the best of our knowledge, investigations of possible high Pi-tolerant AMF species have never been carried out. Perhaps the elaboration of high-resolution markers specific to the Pi^+^ phenotype could help in the detection of Pi-tolerant lines in natural and managed ecosystems, and could improve our knowledge of AMF ecology. At least, our results could suggest that adaptation to higher Pi concentrations of *R. irregulare* could occur in managed conventional systems, and, if true, would give more weight to the negative influence of other factors.

#### 4.4.2 Mycorrhizal production strategies: a step backwards in the definition of a good inoculum?

Our data suggest that propagule abundance may not be the sole determinant for achieving beneficial effects on crops. Traditionally, the production of propagule-rich inocula has been pursued to increase the capacity for inoculating larger areas for commercial purposes and to meet quality criteria for scientific usage. An inoculum with fewer propagules would require higher doses to maintain a minimal but efficient number of propagules applied per plant. As a result, larger quantities would need to be produced, resulting in higher costs for customers and growers. However, the application of elevated Pi concentration during the first generation of *ex vitro* production of RiQS81-Pi^+^ significantly reduced its ability to produce propagules compared to the classical production route of RiQS81-Pi^-^. Interestingly, the difference between the adapted and non-adapted *R. irregulare* gradually decreased over subsequent generations. This suggests that in the future, it may be possible to achieve a similar number of propagules between the two *R. irregulare* phenotypes during production under contrasting Pi fertilization, providing definitive evidence of short-term Pi adaptation.

Conversely, all other things being equal, it is possible that production systems that typically involve specific fertilization, especially low phosphate fertilization, may adapt mycorrhizal species to produce high amounts of propagules, but could potentially lead to reduced response or specific loss of native adaptive traits to a given soil ecosystem. Furthermore, previous studies have shown that the application of exogenous mycorrhizal strains can lead to the genetic replacement of native fungal species and result in limited plant responses (Calvente et al., 2004; Schwartz et al., 2006; Rowe et al., 2007; Estrada et al., 2013; Emam, 2016). It is possible that this limitation is due to the fertilization used during mycorrhizal production, which would make mycorrhizal strains or isolates less able to thrive in certain soil conditions or lead to incompatibilities between exogenous and native species (Bencherif et al., 2023). Our results open the possibility of producing innovative and generic mycorrhizal inocula that could be better adapted to specific fertilization regimes or elemental concentrations.

#### 4.4.3 Consequences of fertilization during inoculum production: what is the right fungal model for research purposes?

Building on the points raised in the previous paragraph, it is logical to address a potential concern about the design of inoculum production and its consequence for scientific purposes. The effects of high phosphate concentrations on mycorrhizal development and response have been extensively studied for decades. Numerous publications conducted under controlled conditions have consistently demonstrated the inhibition of mycorrhizal colonization by phosphate, often associated with negative MGR (Mosse, 1973; Thomson et al., 1986; Smith and Read, 2008; Nagy et al., 2009; Breuillin et al., 2010; Balzergue et al., 2011). Over time, these studies have delved deeper into the topic, using increasingly sophisticated tools such as transcriptomics, genetics, and mutant plants to gain a better understanding of the role of phosphate in endomycorrhizal symbioses. However, it is worth questioning whether we have generated artificial knowledge by using artificially domesticated fungal inocula. Our data challenge the notion that the phenomenon of phosphate inhibition is solely the result of a top-down mechanism in which the plant controls the fungus. Instead, we emphasize the importance of fungal respiration and phosphate metabolism, which play a role in the fungus’ own phenotypic behavior and may also have consequences for the plant response. It is important to note that the mycorrhizal inocula used in previous studies were primarily derived from pot culture systems typically produced by laboratories, companies, or glomeromycotan banks. These systems often include specific phosphate fertilization regimes as a widely used standard for optimal fungal propagation (Ijdo et al., 2011). The production of mycorrhizal fungi under such specific fertilization regimes over multiple generations could have profound effects on fungal physiology, the extent of which is largely unknown when these inocula are used for research purposes. This could potentially explain or contribute to discrepancies in the interpretation of the effects of compounds that influence mycorrhizal behavior, such as hormones or nutrients. Therefore, it will become critical to consider how a fungal inoculum used in research is produced, as it may have important implications for the results and interpretation.

## 5 Conclusion

We provide the first evidence that it is possible to artificially induce short-term adaptation of *R. irregulare* strain QS81 to high phosphate levels. After five *in vitro* and one *ex vitro* generation, this process resulted in differences in phenotypic development, plant responses, and fungal and plant transcriptomic patterns. Our study demonstrates another level of the extreme plasticity that AMF can harbor. The fungal material can be used to provide another basis for the concept of adaptive evolution in Glomeromycota and to unravel its mechanisms under controlled conditions. Moreover, such short-adapted AMF strains could provide a new strategy for the application of specific mycorrhizal inocula in plant production systems and pave the way to elaborate a biotechnological response in crops to associate mycorrhizal fungi where high phosphate fertilization is the standard.

## Data availability statement

The original contributions presented in the study are included in the article/**Supplementary Material**. Further inquiries can be directed to the corresponding authors.

## Author contributions

EL-M: Data curation, Formal analysis, Investigation, Validation, Writing – original draft. LM: Data curation, Formal analysis, Investigation, Validation, Writing – original draft. AJ: Investigation, Writing – review & editing. CS: Funding acquisition, Project administration, Resources, Writing – review & editing. PF: Conceptualization, Funding acquisition, Project administration, Resources, Writing – review & editing.
