# Supplementary material for: Short-term artificial adaptation of Rhizoglomus irregulare to high phosphate levels and its implications for fungal-plant interactions: phenotypic and transcriptomic insights
Source: Front Plant Sci. 2024 Apr 23;15:1385245. doi: 10.3389/fpls.2024.1385245 (PMC11074448; doi:10.3389/fpls.2024.1385245)
Supplement: Supplementary file 3 [file DataSheet_1.pdf]

Short-term artificial adaptation of *Rhizoglomus irregulare* to high phosphate levels and its implications for fungal-plant interactions: phenotypic and transcriptomic insights

Eva Lucic-Mercy[1]*, Louis Mercy[1], Andrea Jeschke[1], Carolin Schneider[1], Philipp Franken[2,3]

[1]INOQ GmbH, Schnega, Germany

[2]Institute of Microbiology, Friedrich Schiller University, Jena, Germany

[3]Erfurt Research Centre for Horticultural Crops, University of Applied Sciences Erfurt, Erfurt, Germany

*** Correspondence:**
Eva Lucic-Mercy
lucic@inoq.de

1 Supplementary methods

1.1 Supplementary method 1: RFLP/sequencing

Genomic DNA was extracted from root powder containing *R. irregulare* QS81 used as source inoculum, respectively Pi^+ and Pi^- phenotypes; from *in vitro* spores of RiQS81 as positive control and from spores of *R. irregulare* isolate A, not present in the laboratory during the trial, as negative control. DNA were extracted using the innuPREP Plant DNA Kit (Innuscreen GmbH, Germany), according to the manufacturers' instructions.

Sequencing of nuclear LSU. A nested PCR was performed. The primer pair ITS1-FLR2 was used for the first PCR reaction (White et al., 1990; Trouvelot et al., 1999). The primer pair FLR2-LROR (Vilgalys and Hester, 1990; Trouvelot et al., 1999) was used in the second PCR reaction. DNA was amplified with ROTI®Pol ProofRead polymerase according to the manufacturers' instructions. The PCR products were purified using the GFX PCR DNA and Gel Band Purification Kit (Cytiva, USA) prior to sequencing (Eurofins genomics, Germany).

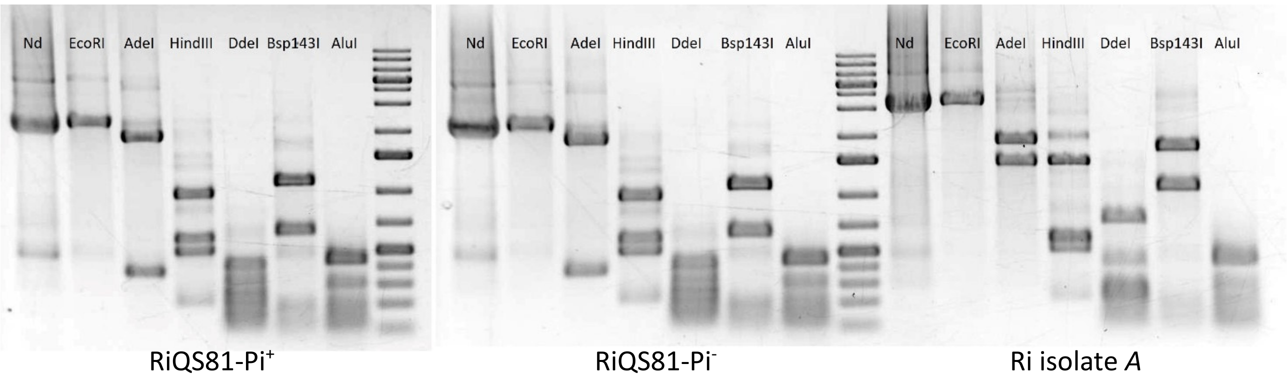

Figure S1. Banding patterns of *R. irregulare* QS81 (Pi^+ and Pi^- phenotypes) and *R. irregulare* isolate A restriction fragment length polymorphism (RFLP) types. Regions of the mitochondrial rRNA large subunit gene (mtLSU) were amplified using nested primers. PCR products were digested with six different restriction enzymes and analyzed by agarose gel electrophoresis. Nd = non digested.

PCR amplification of mtLSU and RFLP. All DNA were amplified by nested PCR as described in Börstler et al. (2008). Forward and reverse primers RNL-28a and RNL-5, respectively, were used in the first reaction and RNL-29 and RNL-30, respectively, in the second reaction. For RFLP analyses, 20 U of the following enzymes: *Eco*RI, *Dra*III, *Hind*III, *Dde*I, *Bsp*143I and *Alu*I (ThermoFisher Scientific, USA) were used per sample. For each reaction 5 µl of the final nested PCR products were digested overnight at 37°C in a total volume of 15 µl. For visualization, 1.5% agarose gels were loaded with 5 µl of the digestion products.

Results. RiQS81-P^- and RiQS81-P^+ presented the same banding patterns after digestion of a mitochondrial mtLSU fragment (Figure S1) and identical partial LSU sequence (LROR-FLR2) after sequencing. These results confirmed that both inocula corresponded to the same *R. irregulare* isolate and that no contamination occurred during the production step.

1.2 Supplementary method 2: Black ink staining protocol (modified from Vierheilig et al., 1998).

- Washing step. Roots were well washed with tap water to remove soil/substrate. Roots must be clean with no soil trace. The washed roots are quickly dried on paper towels and disposed in thermoresistant glass pills, taking care not to exceed 2/3 of the volume and without overloading the roots.
- Clearing step. Roots were covered with 10% KOH taking care to spread them well in the solution. The glass pills containing melon roots were then incubated at 80°C during 40 minutes in dry oven. The KOH was then removed, and the roots were well washed with tap water.
- Staining step. Roots were covered with a black ink solution (5% black ink for HP/Lexmark printers + 8% pure acetate in osmosed water), and incubated overnight at room temperature. Roots were then washed well several times with tap water.
- Destaining step. Roots were covered with tap water and incubated at least 2 hours at room temperature prior using them for microscopic analyses.

1.3 Supplementary method 3: ALP staining protocol (modified from Guillemin et al., 1995).

- Fresh washed roots were harvested on ice and digested in a solution of Tris/citric acid (pH9.2, 0.05M) with sorbitol (50 mg/mL) and a mix of cellulase and pectinase (15 U/mL each). Incubation was performed at RT for 2 hours in the dark. The digestion was performed in 2 mL solution in 2 mL Eppendorf tubes. The solution is always freshly prepared and should not be stored more than 3 hours.
- Roots were washed in ultra-pure water, dried quickly with kitchen paper and then placed in a solution of Tris/citric acid (pH 9.2, 0.05 M) with $MgCl_2$ (0.05%), $MnCL_2$ (0.05%), alpha naphtyl (1 mg/mL) and Fast blue RR salt (1 mg/mL) overnight in dark.
- Roots were then washed under tap water and dried quickly with kitchen paper and transferred in glass tubes. Destaining of root is performed with KOH 10% (overnight at RT).
- Root fragments were cut and placed between slide and cover slip with glycerol for microscopic observation.

2 Supplementary figures

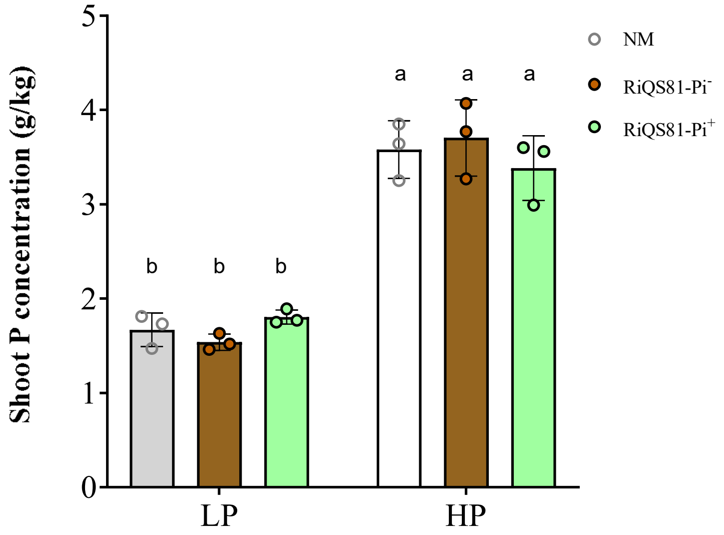

Whole data

Source of Variation	% of total variation	P value	P value summary
Interaction	1.518	0.1970	ns
Phosphate concentration	93.58	<0.0001	****
Inoculation	0.02034	0.9754	ns

Inoculated plant data

Source of Variation	% of total variation	P value	P value summary
Interaction	2.269	0.0973	ns
Phosphate concentration	92.56	<0.0001	****
Pi phenotype	0.01876	0.8687	ns

Figure S2. P content of melon leaves. The P content (g/kg DW) was estimated according to the DIN EN 15621:2017-10 norm (Landesamt für Landwirtschaft und Ländlichen Raum, Thüringen, DE). Data show means (n=3) ± s.e.. Treatments sharing the same letter are not significantly different (p < 0.05 Tukey multiple-comparison ANOVA, SAS Institute Inc., Cary, USA).

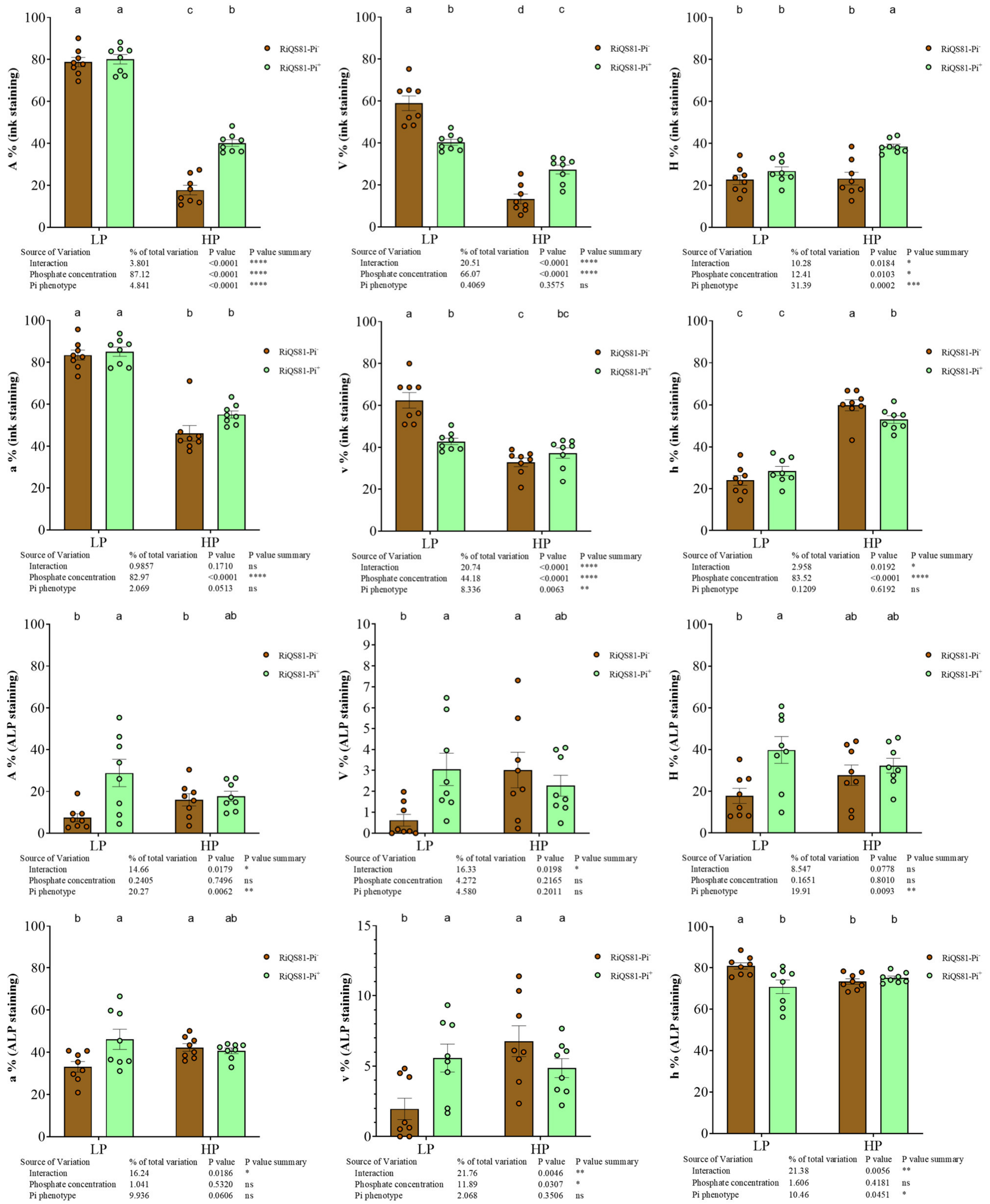

Figure S3. Fungal phenotypic parameters after black ink or ALP staining of RiQS81-Pi$^-$ and RiQS81-Pi$^+$ *R. irregulare* growing within melon roots under two levels of Pi. Roots were harvested 77 DAI, stained and fungal parameter were evaluated. Data show means (n = 8) ± s.e.. Treatments sharing the same letter are not significantly different (< 0.05; Tukey multiple-comparison ANOVA 2 ways), the non-inoculated plants were considered as outgroup to control data normality. Data analyses were performed separately for each parameter. NM: Non-inoculated plants; HP: high phosphate; LP: low phosphate; A%, V% and H%: abundance of arbuscules, vesicles and intraradical mycelium respectively, in the whole root system; a%, v% and h%: abundance of arbuscules, vesicles and intraradical mycelium respectively, in colonized root fragments.

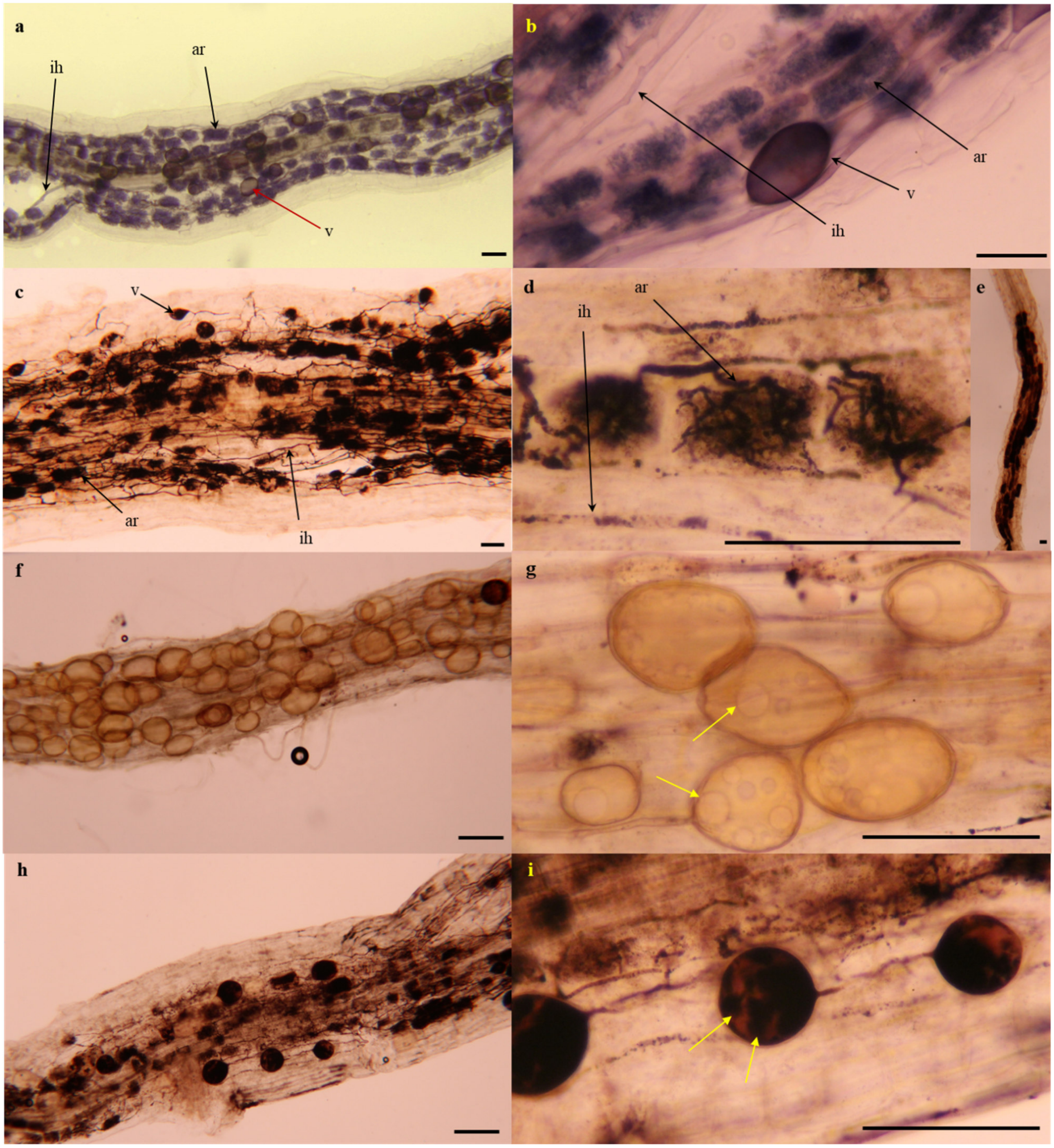

Figure S4. Melon roots inoculated with *R. irregulare* and stained with black (a-b) ink or ALP (c-h). (a-b): low phosphate, RiQS81-Pi^{+}, ink staining; (c-e): high phosphate, RiQS81-Pi^{+}; (f-g): low phosphate, RiQS81-Pi^{-}; (g-h): high phosphate, RiQS81-Pi^{-}. ih: intraradical hyphae, ar: arbuscules, v: vesicle. Yellow arrow: probable lipid droplets inside vesicles. Scale bar: 50 µm.

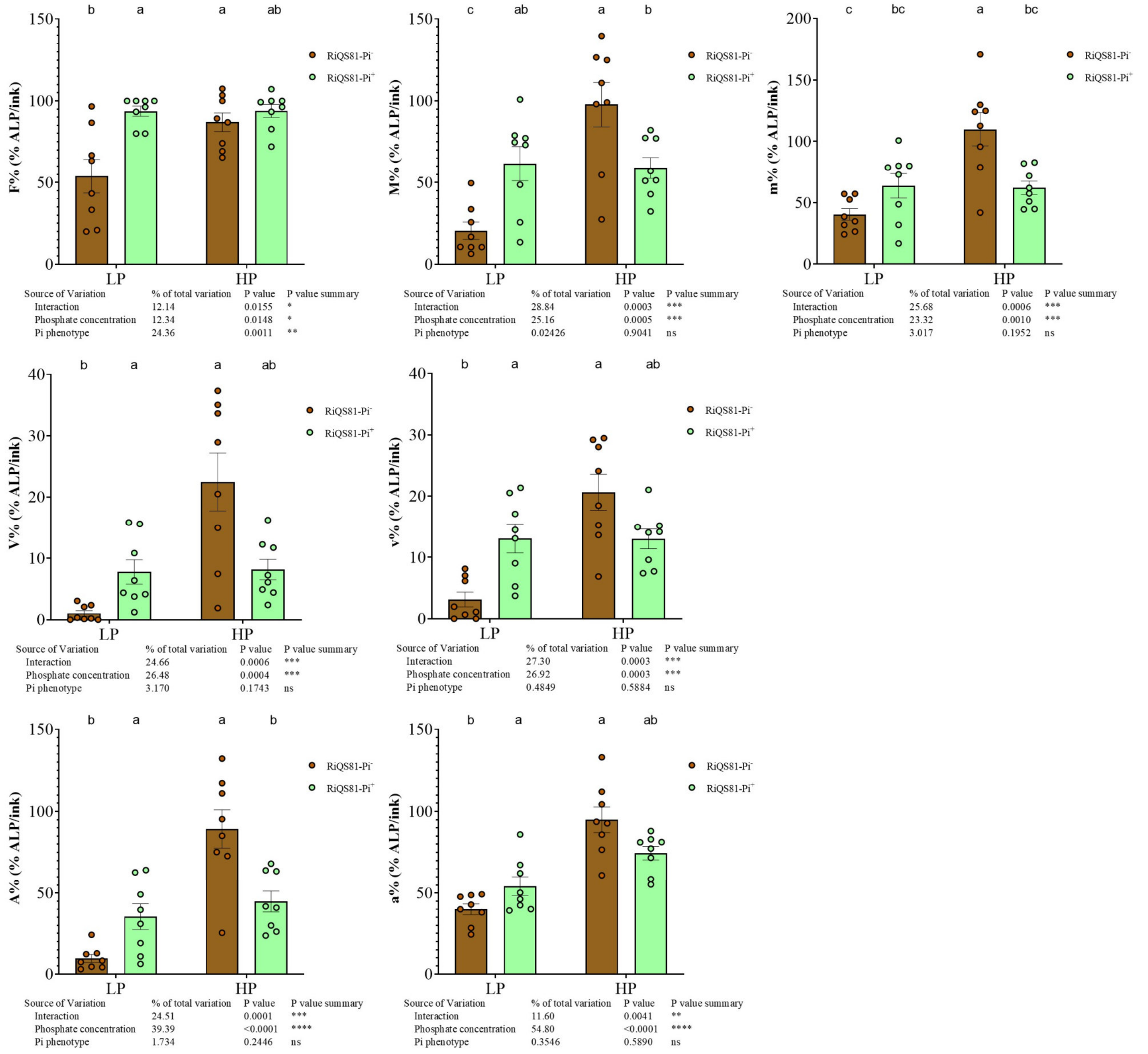

Figure S5. Percentage of ALP-stained structures within ink-stained structures. Data show *means* (n = 8) ± s.e.. Treatments sharing the same letter are not significantly different (P < 0.05; Tukey multiple-comparison ANOVA 2 ways), the non-inoculated plants were considered as outgroup to control data normality. Data analyses were performed separately for each parameter. NM: Non-inoculated plants; HP: high phosphate; LP: low phosphate. F%: frequency of colonized root fragments; M%: intensity of mycorrhizal colonization in the whole root system; m%: intensity of the mycorrhizal colonization in the colonized root fragments; A%, V%: abundance of arbuscules and vesicles respectively, in the whole root system; a%, v%: abundance of arbuscules and vesicles respectively, in mycorrhizal root fragments.

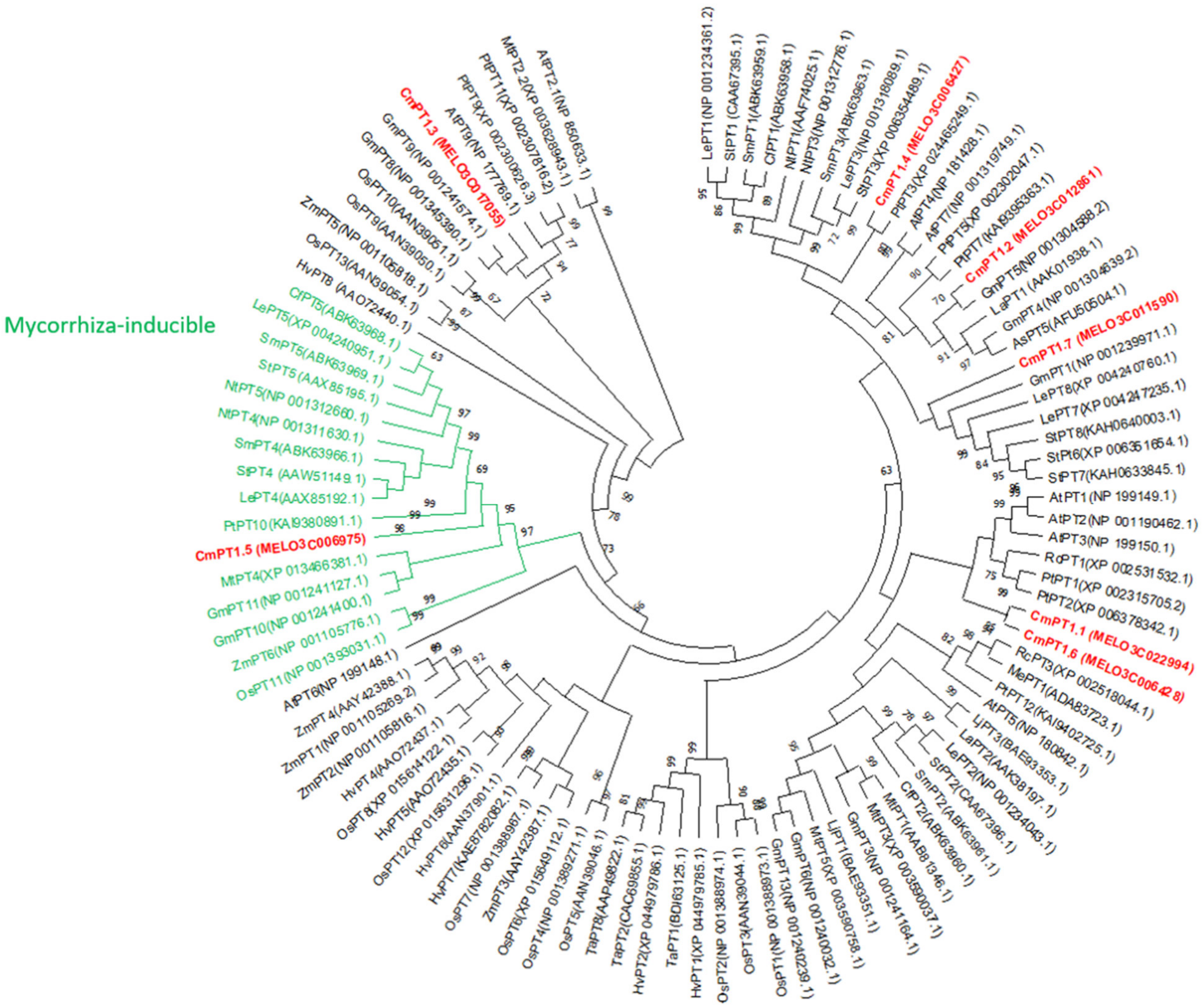

Figure S6. Phylogenetic relationships among melon phosphate transporters (highlighted in red) and already characterized plant sequences belonging to the PHT1 family. The unrooted phylogenetic tree was built using the neighbour-joining method (MEGA11 Molecular Evolutionary Genetics Analysis version 11; Tamura et al., 2021). Bootstraps were performed using 1000 replicates. Protein ID are indicated, either as GeneBank accessions, or according to the Cucurbit Genomics Database (CuGenDBv2; Yu et al., 2023).

LP NM	LP RiQS81-Pi-	LP RiQS81-Pi+	HP NM	HP RiQS81-Pi-	HP RiQS81-Pi+	
0.04	1.61	1.16	-0.88	-0.83	0.02	***CmPT1;1***
-8.07	-7.92	-8.81	-9.69	-9.46	-9.16	***CmPT1;2***
-2.59	-0.63	-1.28	-3.00	-3.00	-2.03	***CmPT1;3***
-2.20	0.47	0.17	-3.36	-3.21	-1.71	***CmPT1;4***
-5.06	4.24	4.75	-10.40	2.25	3.10	***CmPT1;5***
0.02	0.90	-0.09	-2.57	-1.72	-1.85	***CmPT1;6***
-8.18	-7.86	-8.30	-10.06	-9.87	-9.24	***CmPT1;7***

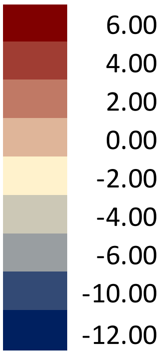

Figure S7. Expression patterns of melon P transporter genes belonging to the PHT1 family. The map was generated using the normalized expression values (dCq). LP: low phosphate; HP: high phosphate; NM: non-mycorrhizal.

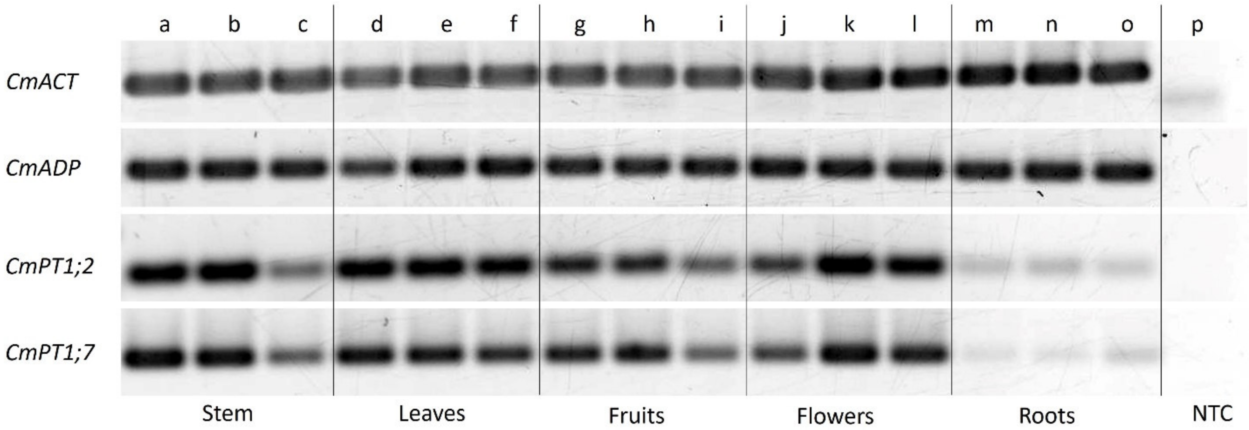

Figure S8. Expression patterns of *CmPT1;2* and *CmPT1;7* through different organs of melon plant. through different organs of melon plant. Total RNA was extracted from 100 mg of melon roots, stem, leaves, flowers, and fruits using the RNeasy Plant Mini Kit (Qiagen, Germany), according to manufacturer's instructions. 60 ng of extracted RNA were reverse transcribed into cDNA using the RevertAid RT Kit (Thermo Fisher Scientific, Waltham, MA, USA) in 20 µL following the supplier's instruction. Tree independent biological replicates were analyzed per plant organ and the PCR reaction was performed with the ROTI®Pol TaqHY polymerase (Carl Roth, Germany). (a - c): Stem; (d- f): Leaves; (g - h): Fruits; (j – l): Flowers; (m – o): roots; (p): non-template control.

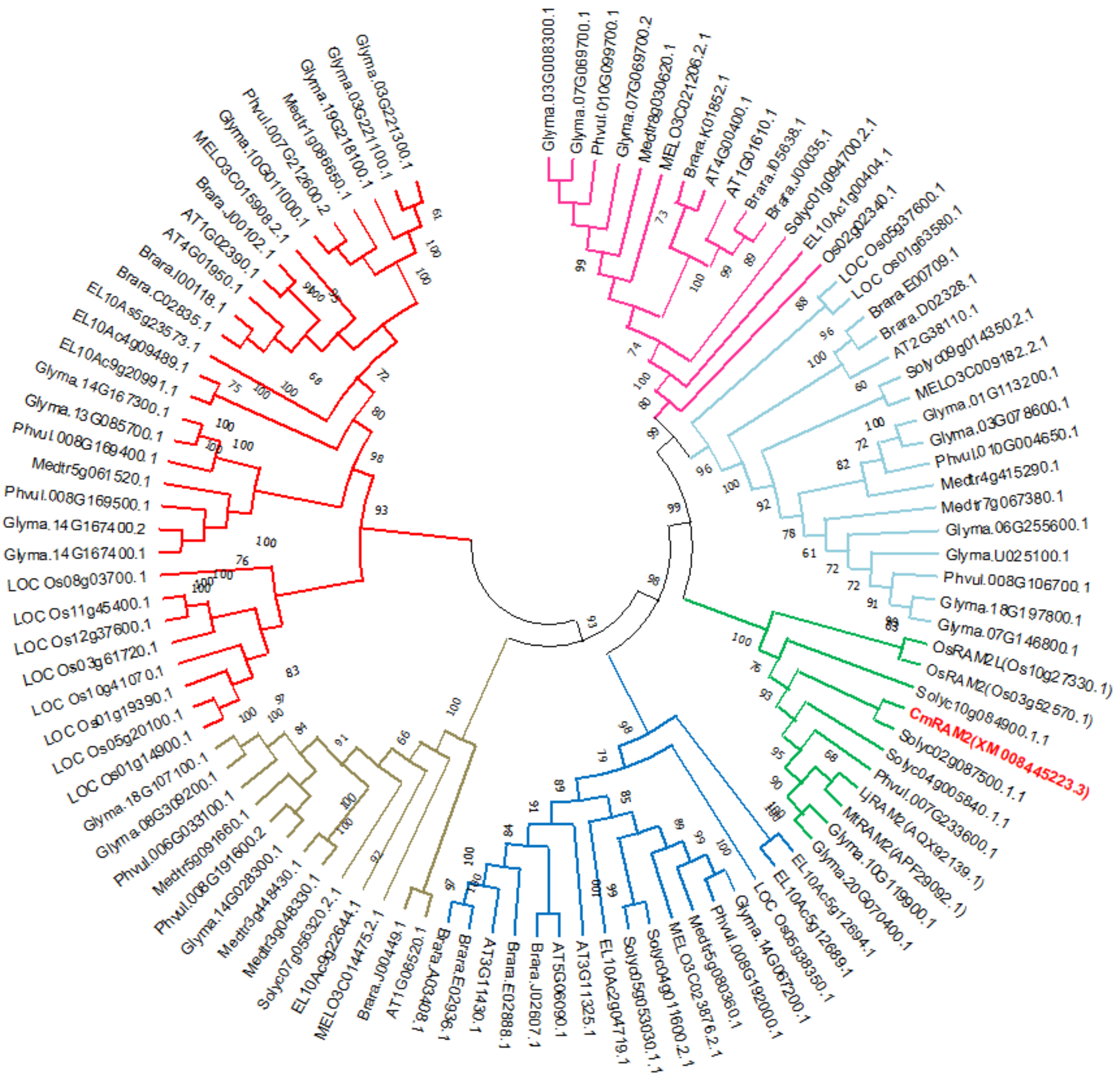

Figure S9. Phylogenetic relationship among plant GPAT sequences. The unrooted phylogenetic tree was built using the neighbour-joining method (MEGA11 Molecular Evolutionary Genetics Analysis version 11; Tamura et al., 2021). Bootstraps were performed using 1000 replicates. The plant species are as follows: *Medicago truncatula* (Medtr); *Solanum lycopersicum* (Soly); *Glycine max* (Glyma); *Arabidopsis thaliana* (AT); *Oryza sativa* (LOC.Os); *Phaseolus vulgaris* (Phvu); *Brassica rapa* (Brara); *Beta vulgaris* (EL). The ID refers to Genbank or Pytozome (https://phytozome-next.jgi.doe.gov/). The different clades were defined according to Liu et al., (2022).

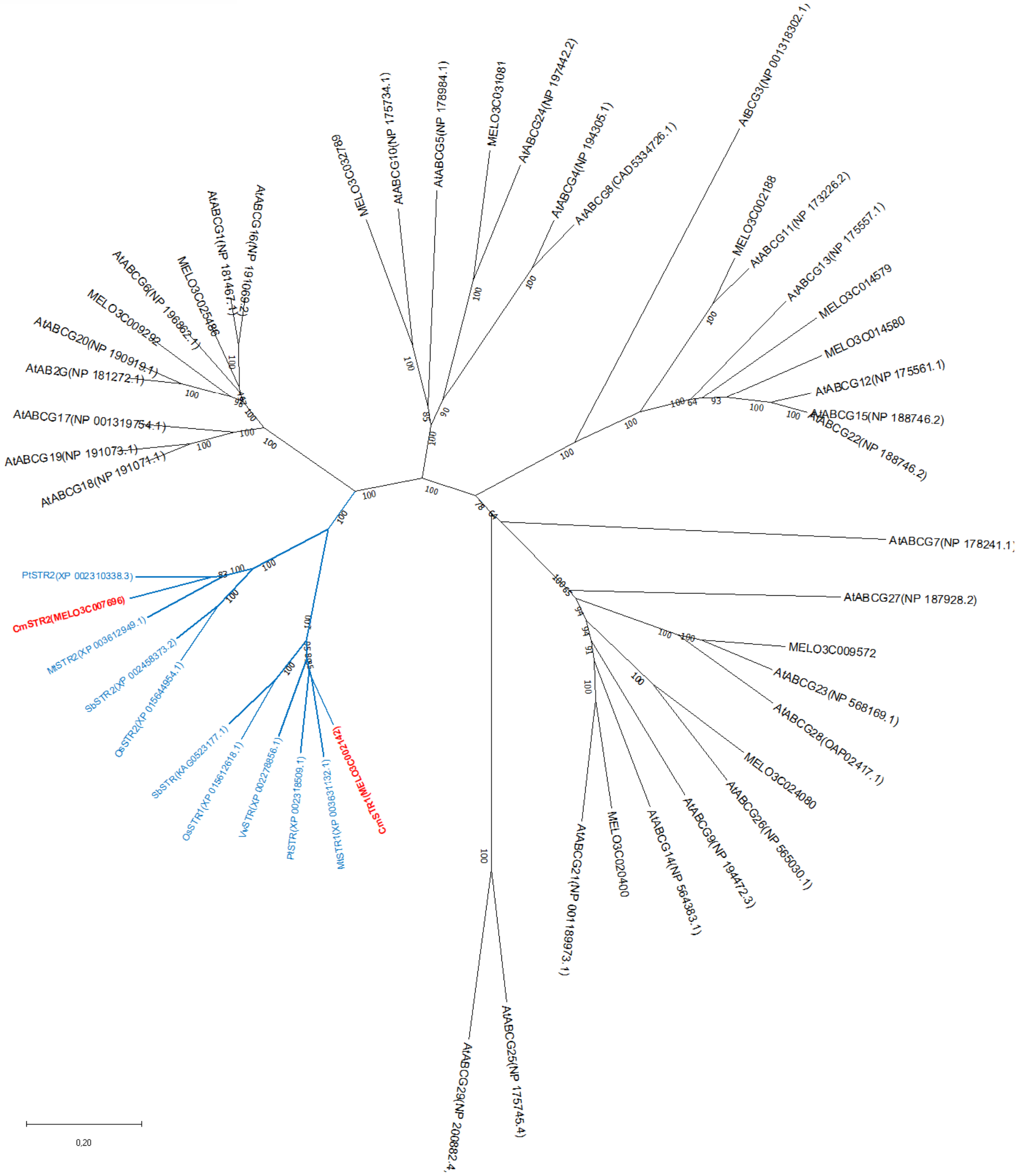

Figure S10. Phylogenetic relationships among melon sequences (highlighted in red) and already characterized plant sequences belonging to the ABCG family. The unrooted phylogenetic tree was built using the neighbour-joining method (MEGA11 Molecular Evolutionary Genetics Analysis version 11; Tamura et al., 2021). Bootstraps were performed using 1000 replicates. Sequences as follows: *Arabidopsis thaliana* (At); *Medicago truncatula* (Mt); *Vinus vitifera* (Vv); *Oryza sativa* (Os); *Sorghum bicolor* (Sb); *Cucumis melo* (Cm/MELO); *Populus trichocarpa* (Pt).

RiQS81-Pi- (LP)	RiQS81-Pi+ (LP)	RiQS81-Pi- (HP)	RiQS81-Pi+ (HP)	
2.40	3.20	2.19	2.88	*RiPT1*
0.07	1.60	-0.38	-0.37	*RiPT2*
-2.53	-2.14	-2.70	-2.21	*RiPT3*
-1.87	-1.64	-1.91	-1.79	*RiPT4*
-0.96	-0.38	-1.02	-0.91	*RiPT5*
-0.54	-0.66	-1.79	-1.79	*RiPT6*
1.44	1.93	1.00	1.00	*RiPT7*
0.19	0.25	0.05	-0.36	*RiSYG1*
2.18	3.77	3.19	3.56	*RiPho1*

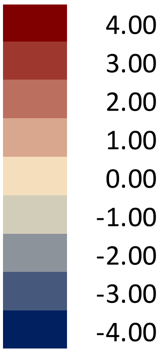

Figure S11. Expression patterns of *R. irregulare* P transporter genes. The map was generated using the normalized expression values (dCq). LP: low phosphate; HP: high phosphate.

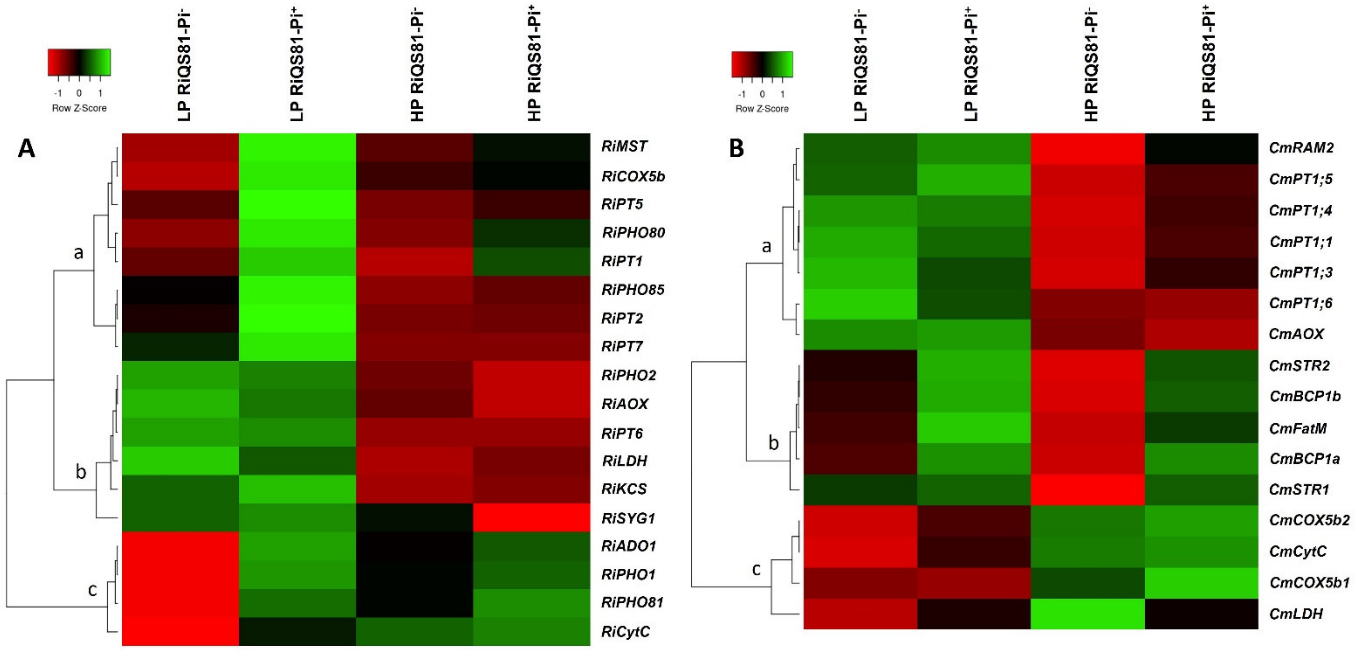

Figure S12. Gene expression clustering for *R. irregulare* (A) and *C. melo* (B) genes. The dendrogram was generated based on dCq values and using the Heatmapper web tool (Babicki et al., 2016). In the fungus (A), the expression of genes can be sorted as those only upregulated in LP Pi^{+} (a), those responded only to Pi concentration (b), and those whose expression was lower only in LP Pi^{-} (c). In the plant (B), the gene expression patterns can be grouped into those that responded to inoculation but whose expression levels followed the colonization rate (a), those for which inoculation with the *R. irregulare* Pi phenotype restored in HP an expression level similar to LP, independently from the mycorrhization rate (b), and those that responded mainly to Pi concentration (c).

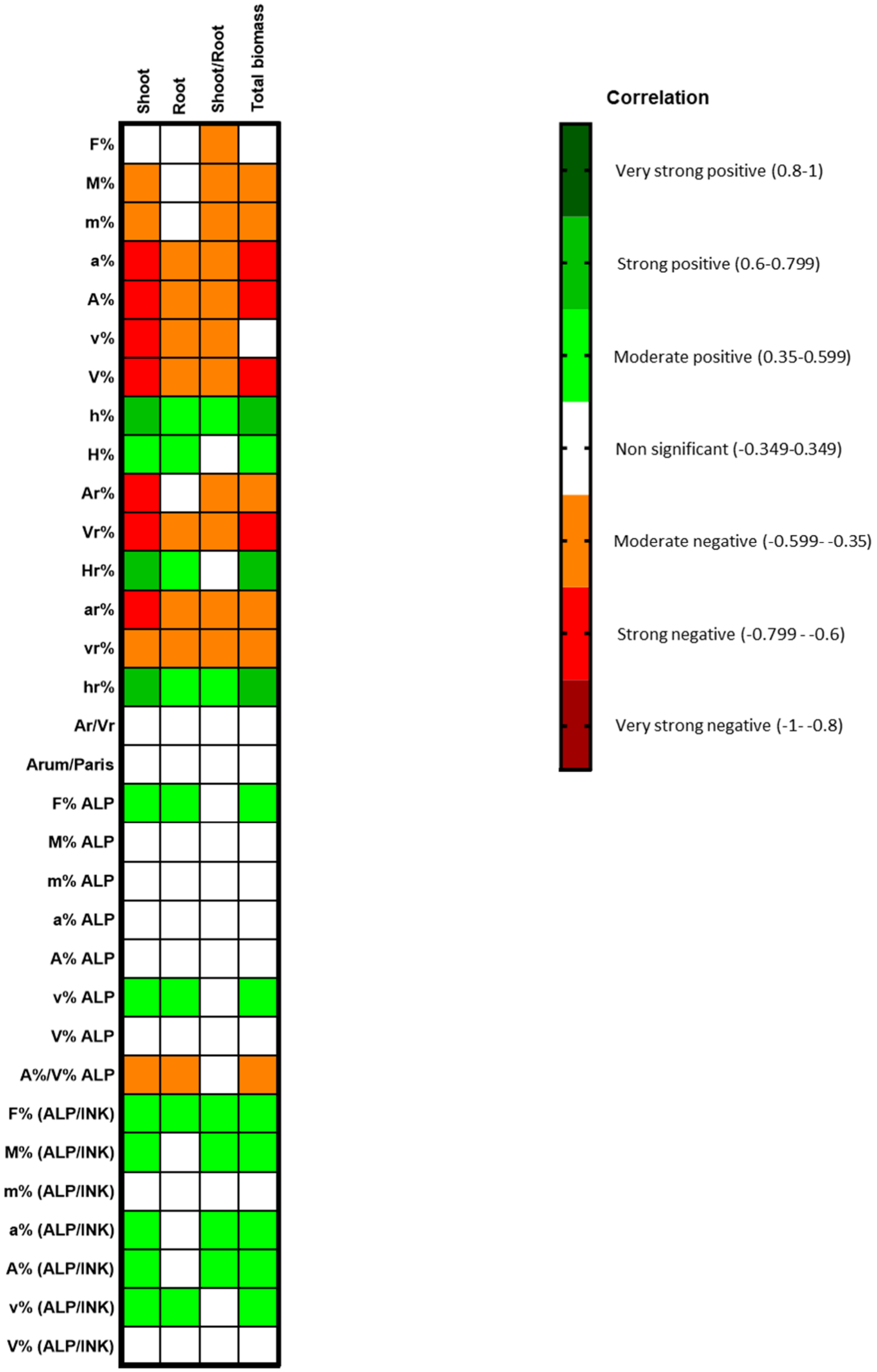

Figure S13. Pearson's correlation heat map for fungal colonization parameters against plant biomass (FW) parameters.

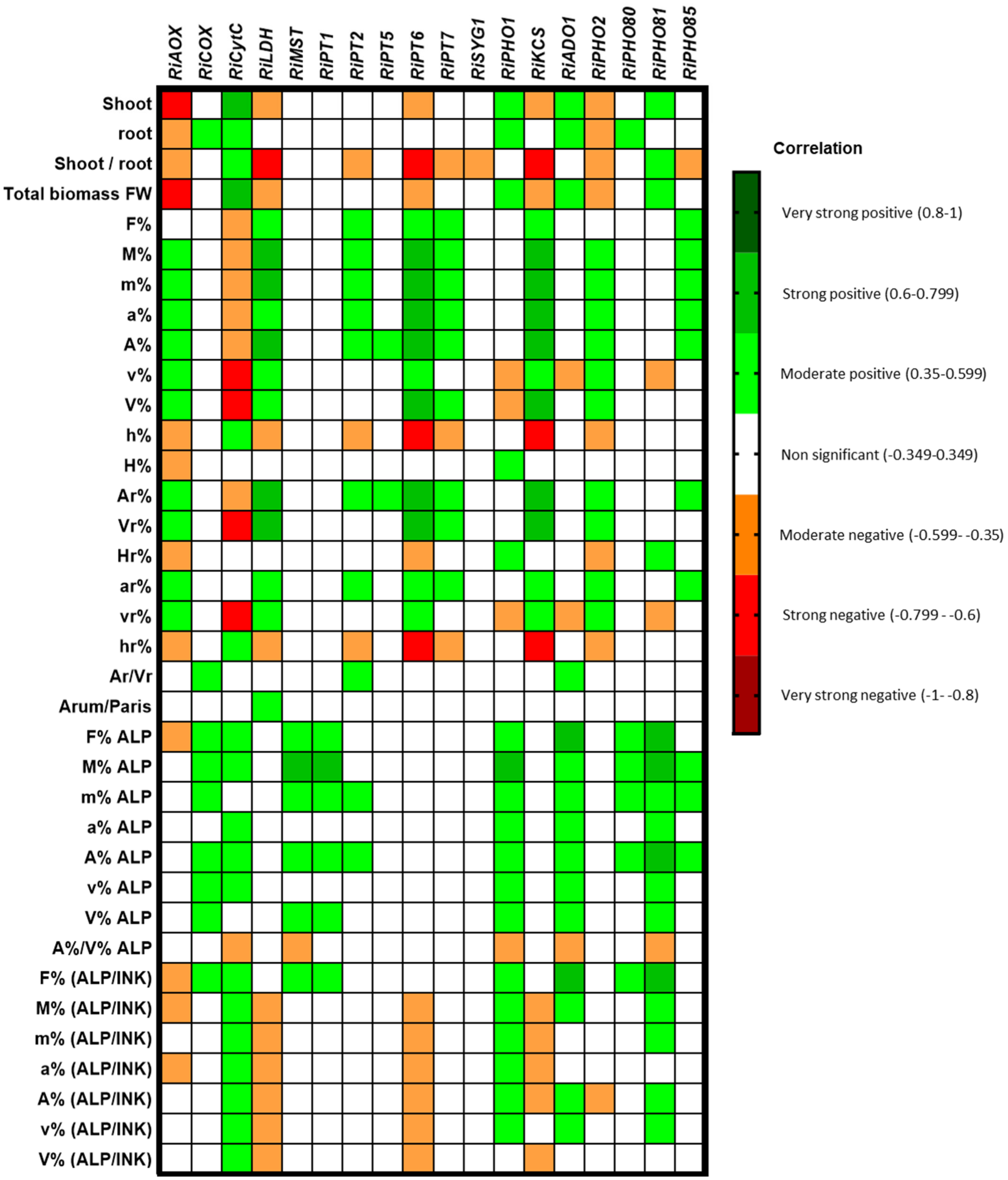

Figure S14. Pearson's correlation heat map for fungal colonization parameters and plant biomass (FW) against fungal transcripts (dCq).

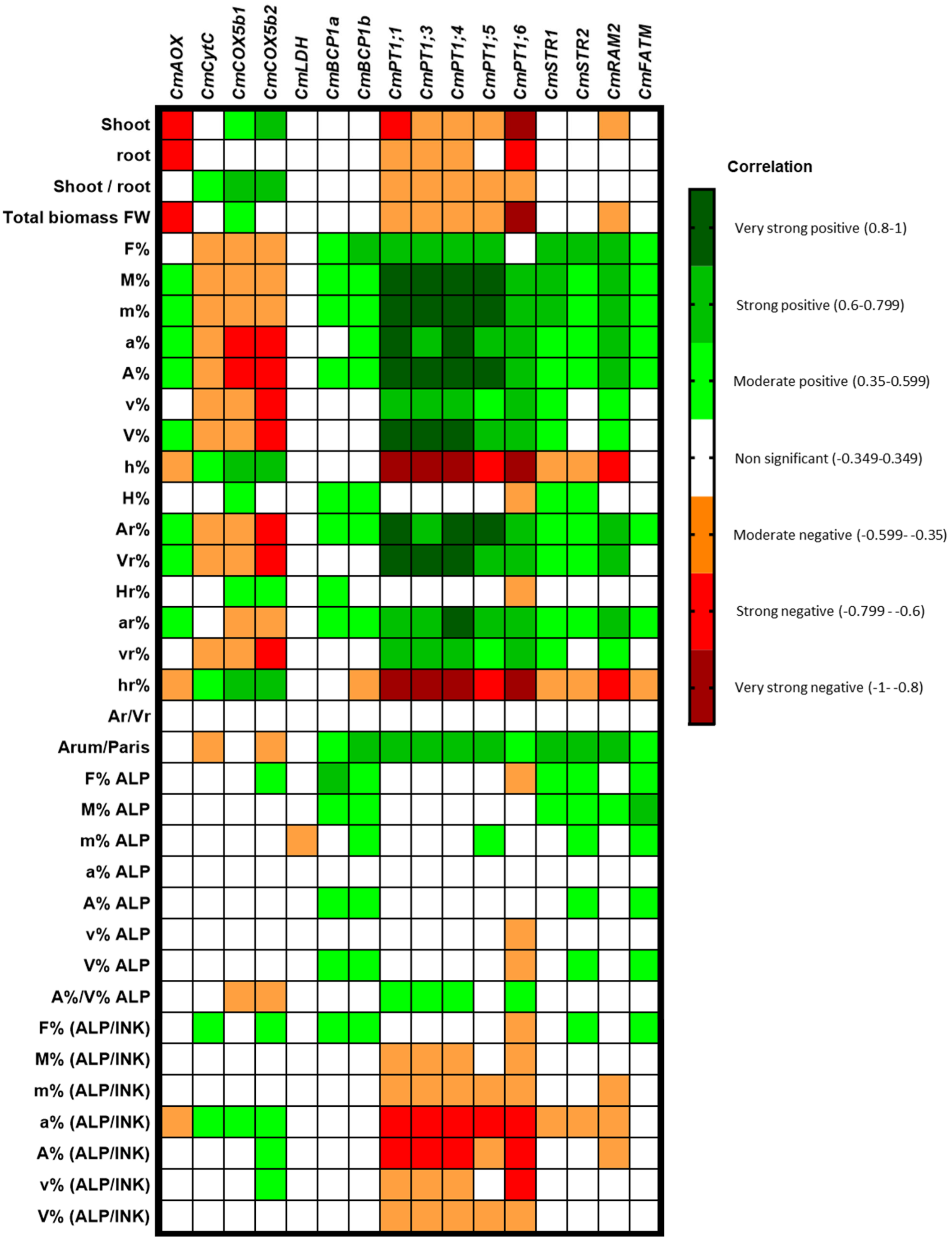

Figure S15. Pearson's correlation heat map for fungal colonization parameters and plant biomass (FW) against plant transcripts (dCq).

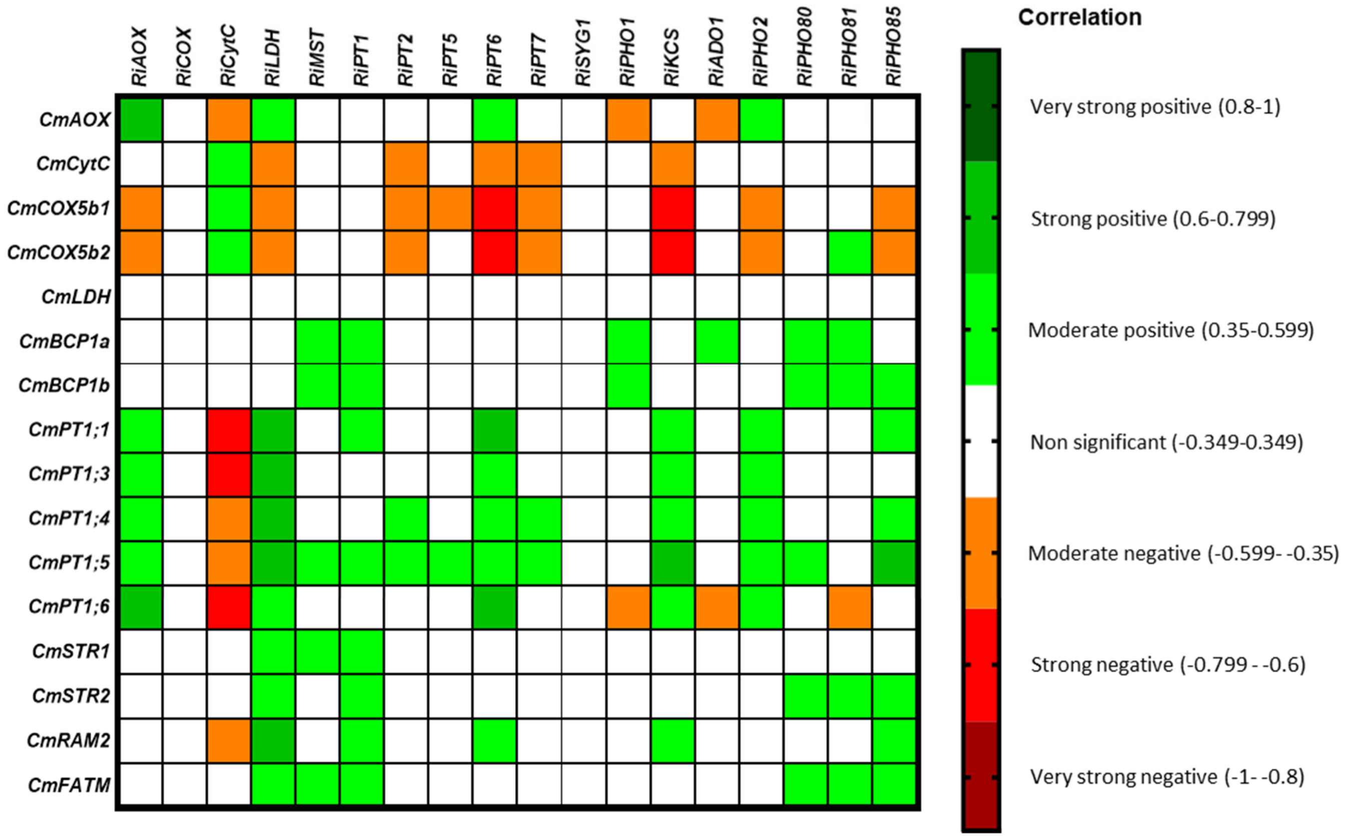

Figure S16. Pearson's correlation heat map for fungal transcripts (dCq) against plant transcripts (dCq).

3 Supplementary table

Table S1. Primers used in this study

Name	Function	Accession	Primer sequence 5'-3	Reference of the primers
RiICL	Isocitrate lyase	XM_025321022.1	F: GCTATTTTGATCATTGCCGCC<br>R: TCATTAAAACGTTCTTCCGACC	Lammers et al., 2001)
RiCOX5b	Cytochrome oxidase, subunit 5b	XM_025309284.1	F: TTGTCGGCTGTACTGGGTTC<br>R: ACCACATTCAGGGCATCTGT	Mercy et al., 2017
RiCytC	Cytochrome C	XM_025309081	F: GCTGCAAGTGCTGATGCTAA<br>R: GGAACCTCCTGCTTCGACTG	Mercy et al., 2017
RiAOX	Alternative oxidase	KT423114.1a	F: AAAATGAACGTATGCACTTGATGAC<br>R: GCGTTCCCACCAGGTAGGT	Mercy et al., 2017
RiLDH	Lactate dehydrogenase	XM_025309035.1	F: CGCTGTAATTGGAGCTGGTT<br>R: TTGACCAGCCTCTTGGAAAG	Mercy et al., 2017
RiPT1	Plasma membrane phosphate transporter	XM_025315184.1	F: AACACGATGTCAACAAAGCAAC<br>R: AAGACCGATTCCATAAAAAGCA	Fiorilli et al., 2013
RiPT2		XM_025313512.1	F: ATTGCTATGGGCGCCTCTTT<br>R: TTTTCCGGACGCTGCTGATA	Xie et al., 2022
RiPT3		XM_025309069.1	F: AAAGGCGTGGAGCAATGA<br>R: CGGGAATAATACCGACACCA	Mercy et al., 2017
RiPT4		XM_025323230.1	F: CGCTGGAGTTGTATCTTTTGTTAC<br>R: CGAAAATAAAGTGCGATTGTAGG	Xie et al., 2022
RiPT5		KU219932.1	F: CCGCCCGTAGTGTGAATAAA<br>R: GAAGCGAATGAGGCAGTAAGAAT	Mercy et al., 2017
RiPT6		XM_025317277.1	F: TAGCCTTCGCTGGTGGTTTT<br>R: TGCAGCACCGAATAATGCAC	This study
RiPT7		LC339948.1	F: CCAGTCTCAGGATTCCCAAA<br>R: CCGATCGTGACAACACAAAG	Mercy et al., 2017
RiPHO1		XM_025309663.1	F: TACAATCACCCGCTACTAAAGC<br>R: TAGTTCGCATAAGGACCAATCC	Xie et al., 2022
RiSYG1		XM_025314561.1	F: TTTACCGCAACGTAGAGCAG<br>R: ACTTCGCTGCAAGCATAAGA	Xie et al., 2022
RiMST2	Sugar transporter	XM_025331163.1	F: GGCAGGATATTTGTCTGATAG<br>R: GCAATAACTCTTCCCGTATAC	Zhou et al., 2021
RiPHO2	Phosphate sensing/signalling	XM_025315882.1	F: TCGACAATATGCTCCGTGAAG<br>R: GGGAATAAATTGTGTTGGCGAG	Xie et al., 2022
RiPHO80		XM_025309562.1	F: AGTTACAGCCTCTTCCAAAGC<br>R: TTTCCCCAGAGCAGATCAAC	Zhou et al., 2021
RiPHO81		XM_025320482.1	F: AAAGCTGTCACAATAGGTACTCC<br>R: GATATCATGGTCCTCTTCGCC	Zhou et al., 2021
RiPHO85		XM_025309192.1	F: TCCCTTCCGGTGTAAATATGC<br>R: TGTGAAGGTGCGTTAGAAGG	Zhou et al., 2021
RiKcs1		XP025174322.1	F: CAATCCACGCGACATAGAC<br>R: AAACACATCATCTTCCTCACC	This study
RiAdo1		XP025188792.1	F: CCAAAAGTCAATACAAAACGCC<br>R: CCACCAACAAAAGCATCACC	This study
CmACT	β-actin	MU51303	F: CCTGGTATCGCTGACCGTAT<br>R: TACTGAGCGATGCAAGGATG	Kong et al., 2014
CmADP	ADP-ribosylation factor 1	MU47713	F: ATATTGCCAACAAGGCGTAGA<br>R: TGCCCGTAAACAAGGGATAAA	Kong et al., 2014
CmCOX5b1	Cytochrome oxidase, subunit 5b	XM_008456053.2	F: TCCGGATCCATTTCCACAGC<br>R: GAAGAGCGAAACGCAAGGTG	This study
CmCOX5b2		XM_017044915	F: TTTTCGCTCGCCATTACGCC<br>R: TGCTCATCCTCGTCTTCACCTC	This study
CmCytC	Cytochrome C	XM_008454159.2	F: TGGCAAGCTTTGACGAAGCAC<br>R: ACGTTCCTGCGGTTTCTTCAAC	This study
CmAOX	Alternative oxidase	NM_001319319.1	F: TGGGAGACTTACAGAGCGGA<br>R: TTCTGCTTCTTCCAGCAGGG	This study
CmLDH	Lactate dehydrogenase	XM_008438878.2	F: AAGAGGTCATAGGTGGGGCT<br>R: GTCCCGAAGGATGGAACGAG	This study
CmPT1;1	Plasma membrane phosphate transporter	LN713259.1	F: CGTCGCTTTAATCGTCTCTGCC<br>R: ATCCGCCGCTGCTTGTTTTG	This study
CmPT1;2		XM_008449435.2	F: GCAACAACTTTCGTCGTCCC<br>R: AATGCACCCACCATAGCTCC	This study
CmPT1;3		XM_008454792.2	F: ATTCCCGCCACCGTCAAATC<br>R: TAATCTCCCCCAATTCCCAGCC	This study
CmPT1;4		XM_008440306.2	F: TTACCCACTTTCCGCCACCATC<br>R: TTCGCCACACATAATCCGCC	This study
CmPT1;5		XM_008441180.1	F: GTCCTTCCTGCTGAGCTTTTCC<br>R: TTCATTTTCACCTTCCCTGCCC	This study

Table S1. Primers used in this study (continued)

Name	Function	Accession	Primer sequence 5'-3	Reference of the primers
CmPT1;6	Plasma membrane phosphate transporter	XM_008440307.1	F: AATTCTGGCTGGCGGAATCA<br>R: GCCATACGTAGTCTGCCTCC	This study
CmPT1;7		XM_008447459.2	F: CCGGGTTGGTCGGTTCTTTA<br>R: GTTCCCGGTCCAATGATGGT	This study
CmSTR1	Fatty acid transport and metabolism	XM_008447787.3	F: CAGCAAGAACGCCAGGACAAAC<br>R: GCCACGAAAGCACAATAACCTCAC	This study
CmSTR2		XM_051088815.1	F: ACAGCCACGACATTCCTCCATAC<br>R: CCCAAGCAAACATCACAGCCAC	This study
CmFATM		XM_008468942.3	F: TGATGAACCAGCAAACAAGACGAC<br>R: GCTTCCGCATTCCCTTCTATACTCC	This study
CmRAM2		XM_008445223.3	F: GAGCAGTCCAACAAATTACTCAGCC<br>R: GCAAGCAAGCGAGAACGAATCC	This study
CmBCP1a	Blue copper proteins	XM_008438947.3	F: GTGGAAAAGAAGGCCACTGTGGAC<br>R: CATCATCATCCCAAGGACGGCAAC	This study
CmBCP1b		XM_008439498.1	F: TCTTCCACCAGTGCTTGTCC<br>R: GTTTGGTTCTGAGGAGGCGA	This study

4 References

Babicki, S., Arndt, D., Marcu, A., Liang, Y., Grant, J. R., Maciejewski, A., et al. (2016). Heatmapper: web-enabled heat mapping for all. *Nucleic Acids Res.* 44, W147-153. doi: 10.1093/nar/gkw419.

Börstler, B., Raab, P. A., Thiéry, O., Morton, J. B., and Redecker, D. (2008). Genetic diversity of the arbuscular mycorrhizal fungus Glomus intraradices as determined by mitochondrial large subunit rRNA gene sequences is considerably higher than previously expected. *New Phytol.* 180, 452–465. doi: 10.1111/j.1469-8137.2008.02574.x.

Fiorilli, V., Lanfranco, L., and Bonfante, P. (2013). The expression of GintPT, the phosphate transporter of *Rhizophagus irregularis*, depends on the symbiotic status and phosphate availability. *Planta* 237. doi: 10.1007/s00425-013-1842-z.

Guillemin, J. P., Orozco, M. O., Gianinazzi-Pearson, V., and Gianinazzi, S. (1995). Influence of phosphate fertilization on fungal alkaline phosphatase and succinate dehydrogenase activities in arbuscular mycorrhiza of soybean and pineapple. *Agric. Ecosyst. Environ.* 53, 63–69. doi: 10.1016/0167-8809(94)00555-S.

Helber, N., Wippel, K., Sauer, N., Schaarschmidt, S., Hause, B., and Requena, N. (2011). A Versatile Monosaccharide Transporter That Operates in the Arbuscular Mycorrhizal Fungus Glomus sp Is Crucial for the Symbiotic Relationship with Plants. *Plant Cell* 23, 3812–3823. doi: 10.1105/tpc.111.089813.

Kong, Q., Yuan, J., Niu, P., Xie, J., Jiang, W., Huang, Y., et al. (2014). Screening Suitable Reference Genes for Normalization in Reverse Transcription Quantitative Real-Time PCR Analysis in Melon. *PLoS ONE* 9, e87197. doi: 10.1371/journal.pone.0087197.

Lammers, P. J., Jun, J., Abubaker, J., Arreola, R., Gopalan, A., Bago, B., et al. (2001). The Glyoxylate Cycle in an Arbuscular Mycorrhizal Fungus. Carbon Flux and Gene Expression. *Plant Physiol.* 127, 1287–1298. doi: 10.1104/pp.010375.

Liu, Y.-N., Liu, C.-C., Zhu, A.-Q., Niu, K.-X., Guo, R., Tian, L., et al. (2022). OsRAM2 Function in Lipid Biosynthesis Is Required for Arbuscular Mycorrhizal Symbiosis in Rice. *Mol. Plant. Microbe Interact.* 35, 187–199. doi: 10.1094/MPMI-04-21-0097-R.

Mercy, L. (2017). *INOQ Calculator Advanced. Evaluate the mycorrhizal rate according to a modified Trouvelot method.* doi: 10.13140/RG.2.2.13641.03684.

Mercy, L., Lucic-Mercy, E., Nogales, A., Poghosyan, A., Schneider, C., and Arnholdt-Schmitt, B. (2017). A Functional Approach towards Understanding the Role of the Mitochondrial Respiratory Chain in an Endomycorrhizal Symbiosis. *Front. Plant Sci.* 8. doi: 10.3389/fpls.2017.00417.

Tamura, K., Stecher, G., and Kumar, S. (2021). MEGA11: Molecular Evolutionary Genetics Analysis Version 11. *Mol. Biol. Evol.* 38, 3022–3027. doi: 10.1093/molbev/msab120.

Trouvelot, A., Kough, J. L., and Gianinazzi-Pearson, V. (1986). "Mesure du taux de mycorhization VA d'un système radiculaire. Recherche de méthode d'estimation ayant une signification fonctionnelle," in *Physiological and genetical aspects of mycorrhizae : proceedings of the 1st european symposium on mycorrhizae, Dijon, 1-5 July 1985.*, 217–221.

Trouvelot, S., van Tuinen, D., Hijri, M., and Gianinazzi-Pearson, V. (1999). Visualization of ribosomal DNA loci in spore interphasic nuclei of glomalean fungi by fluorescence in situ hybridization. *Mycorrhiza* 8, 203–206. doi: 10.1007/s005720050235.

Vierheilig, H., Bago, B., Albrecht, C., Poulin, M.-J., and Piché, Y. (1998). "Flavonoids and Arbuscular-Mycorrhizal Fungi," in *Flavonoids in the Living System* Advances in Experimental Medicine and Biology., eds. J. A. Manthey and B. S. Buslig (Boston, MA: Springer US), 9–33. doi: 10.1007/978-1-4615-5335-9_2.

Vilgalys, R., and Hester, M. (1990). Rapid genetic identification and mapping of enzymatically amplified ribosomal DNA from several Cryptococcus species. *J. Bacteriol.* 172, 4238–4246.

White, T. J., Bruns, T., Lee, S., and Taylor, J. (1990). "Amplification and direct sequencing of fungal ribosomal RNA genes for phylogenetics," in *PCR Protocols: A Guide to Methods and Applications* (Academic Press), 315–322. doi: 10.1016/B978-0-12-372180-8.50042-1.

Xie, X., Lai, W., Che, X., Wang, S., Ren, Y., Hu, W., et al. (2022). A SPX domain-containing phosphate transporter from *Rhizophagus irregularis* handles phosphate homeostasis at symbiotic interface of arbuscular mycorrhizas. *New Phytol.* 234, 650–671. doi: 10.1111/nph.17973.

Yu, J., Wu, S., Sun, H., Wang, X., Tang, X., Guo, S., et al. (2023). CuGenDBv2: an updated database for cucurbit genomics. *Nucleic Acids Res.* 51, D1457–D1464. doi: 10.1093/nar/gkac921.
